# Elimination of aromatic fusel alcohols as by-products of *Saccharomyces cerevisiae* strains engineered for phenylpropanoid production by 2-oxo-acid decarboxylase replacement

**DOI:** 10.1016/j.mec.2021.e00183

**Published:** 2021-09-07

**Authors:** Else-Jasmijn Hassing, Joran Buijs, Nikki Blankerts, Marijke A. Luttik, Erik A.de Hulster, Jack T. Pronk, Jean-Marc Daran

**Affiliations:** Department of Biotechnology, Delft University of Technology, van der Maasweg 9, 2627 HZ, Delft, the Netherlands

**Keywords:** Pyruvate decarboxylase, Phenylpyruvate decarboxylase, *Saccharomyces cerevisiae*, Ehrlich pathway, Fusel alcohols, Phenylpropanoid

## Abstract

Engineered strains of the yeast *Saccharomyces cerevisiae* are intensively studied as production platforms for aromatic compounds such as hydroxycinnamic acids, stilbenoids and flavonoids. Heterologous pathways for production of these compounds use l-phenylalanine and/or l-tyrosine, generated by the yeast shikimate pathway, as aromatic precursors. The Ehrlich pathway converts these precursors to aromatic fusel alcohols and acids, which are undesirable by-products of yeast strains engineered for production of high-value aromatic compounds. Activity of the Ehrlich pathway requires any of four *S. cerevisiae* 2-oxo-acid decarboxylases (2-OADCs): Aro10 or the pyruvate-decarboxylase isoenzymes Pdc1, Pdc5, and Pdc6. Elimination of pyruvate-decarboxylase activity from *S. cerevisiae* is not straightforward as it plays a key role in cytosolic acetyl-CoA biosynthesis during growth on glucose. In a search for pyruvate decarboxylases that do not decarboxylate aromatic 2-oxo acids, eleven yeast and bacterial 2-OADC-encoding genes were investigated. Homologs from *Kluyveromyces lactis* (*KlPDC1*), *Kluyveromyces marxianus* (*KmPDC1*), *Yarrowia lipolytica* (*YlPDC1*), *Zymomonas mobilis* (*Zmpdc1*) and *Gluconacetobacter diazotrophicus* (*Gdpdc1.2* and *Gdpdc1.3*) complemented a Pdc^−^ strain of *S. cerevisiae* for growth on glucose. Enzyme-activity assays in cell extracts showed that these genes encoded active pyruvate decarboxylases with different substrate specificities. In these *in vitro* assays, *Zm*Pdc1, *Gd*Pdc1.2 or *Gd*Pdc1.3 had no substrate specificity towards phenylpyruvate. Replacing Aro10 and Pdc1,5,6 by these bacterial decarboxylases completely eliminated aromatic fusel-alcohol production in glucose-grown batch cultures of an engineered coumaric acid-producing *S. cerevisiae* strain. These results outline a strategy to prevent formation of an important class of by-products in ‘chassis’ yeast strains for production of non-native aromatic compounds.

## Highlights

• Identification of pyruvate decarboxylases active with pyruvate but not with aromatic 2-oxo acids.
• *Zymomonas mobilis* pyruvate decarboxylase can replace the native yeast enzymes.
• Expression of *Z. mobilis* pyruvate decarboxylase removes formation of fusel alcohols.
• Elimination of fusel alcohol by products improves formation of coumaric acid.
• Decarboxylase swapping is a beneficial strategy for production of non-native aromatics.

## 1 Introduction

The aromatic amino acids l-phenylalanine and l-tyrosine are precursors of many industrially relevant compounds belonging to the phenylpropanoid family of aromatic compounds (Neelam et al., 2020), including hydroxycinnamic acids (Vannelli et al., 2007), stilbenoids (Trantas et al., 2009) and flavonoids (Falcone Ferreyra et al., 2012). These compounds have diverse applications in the food, chemical, pharmaceutical and cosmetic industries (Neelam et al., 2020). Current production processes mostly depend on petroleum-based chemical processes (Das et al., 2007) or direct extraction from plants (Trantas et al., 2015). However, the chemical processes involved are often inefficient and unsustainable (Chemler and Koffas, 2008; Bhan et al., 2013; Zha et al., 2019) while plant extraction processes are limited by biomass availability, low extraction yields and low purity of the final products (Zhang, 2007; Rodriguez et al., 2017). To overcome these pitfalls and meet the increasing demand for biologically and renewably sourced aroma and flavour compounds, microbial production from renewable feedstocks offers a promising alternative (Trantas et al., 2015).

**Fig. 1:**
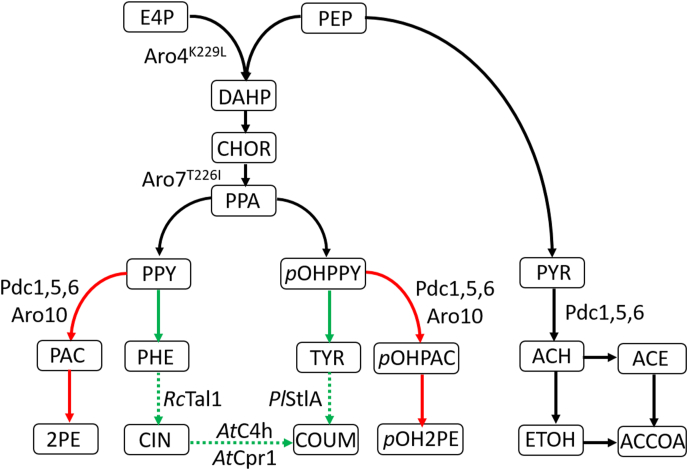
**Metabolic pathways involved in production of the fusel alcohols 2-phenylethanol and *p-*hydroxyphenylethanol and their relation to coumaric acid production in *S. cerevisiae*.** The 2-oxo acids phenylpyruvate and *p*-hydroxyphenylpyruvate can be decarboxylated into the fusel aldehydes phenylacetaldehyde or *p-*hydroxyphenylacetaldehyde, by 2-oxo acid decarboxylases (Pdc1, Pdc5, Pdc6, Aro10) (in red), or be transaminated into l-phenylalanine or l-tyrosine (in green), respectively. These two aromatic amino acids can both serve as substrate in the phenylpropanoid pathway (indicated with dotted arrows), for cinnamic acid and coumaric acid biosynthesis. The 2-oxo acid decarboxylases (Pdc1, Pdc5, Pdc6, Aro10) are also responsible for the decarboxylation of pyruvate into acetaldehyde, a step essential for cytosolic acetyl-CoA biosynthesis. E4P erythose-4-phosphate, PEP phosphoenolpyruvate, DAHP 3-deoxy-D-arabino-heptulosonate-7-phosphate, CHOR chorismate, PPA prephenate, PPY phenylpyruvate, PHE l-phenylalanine, PAC phenylacetaldehyde, 2 PE 2-phenylethanol, *p*OHPPY *p*-hydroxyphenylpyruvate, TYR l-tyrosine, *p*OHPAC *p*-hydroxyphenylacetaldehyde, *p*OH2PE *p*-hydroxyphenylethanol, PYR pyruvate, ACH acetaldehyde, ACE acetate, ETOH ethanol, ACCOA acetyl-CoA.

Development of microbial platforms for *de novo* production of aromatic compounds has been intensively studied in the yeasts *S. cerevisiae* and *Y. lipolytica* (Liu et al., 2020). The yeast shikimate pathway for aromatic amino-acid biosynthesis, a focal point in these metabolic engineering studies, is initiated by condensation of phospho-enol-pyruvate (PEP) and erythrose-4-phosphate (E4P) to form 3-deoxy-d-arabino-heptulosonate-7-phosphate (DAHP). This seven-carbon intermediate is converted into chorismate via a series of biochemical reactions (Fig. 1). At chorismate, the pathway for l-tryptophan synthesis branches off. For biosynthesis of l-phenylalanine and l-tyrosine, chorismate is converted to prephenate, from which either phenylpyruvate or *p*-hydroxyphenylpyruvate are formed. Transamination of these two aromatic 2-oxo acids then yields l-phenylalanine and l-tyrosine, respectively.

In addition to high-level functional expression of heterologous pathway enzymes (Liu et al., 2016, 2019; Yang et al., 2020), elimination of allosteric feed-back inhibition of the shikimate-pathway enzymes DHAP synthase (Aro3 and Aro4) and chorismate mutase (Aro7) (Hartmann et al., 2003, Krappmann et al., 2000, Luttik et al., 2008, Reifenrath and Boles, 2018, Schnappauf et al., 1998), increasing the capacity of the shikimate pathway (Koopman et al., 2012; Paddon et al., 2013; Liu et al., 2016, 2019; Rodriguez et al., 2017; Levisson et al., 2018; Gu et al., 2020; Palmer et al., 2020; Sáez-Sáez et al., 2020; Yang et al., 2020) and improving supply of its precursors PEP and E4P (Liu et al., 2019; Gu et al., 2020; Yang et al., 2020) have enabled increased titers and yields of phenylpropanoid in *S. cerevisiae*. However, these metabolic engineering strategies also lead to increased formation of aromatic fusel alcohols (2-phenylethanol, *p*-hydroxyphenylethanol) (Koopman et al., 2012; Levisson et al., 2018; Liu et al., 2019) and fusel acids (phenylacetic acid, *p*-hydroxyphenylacetic acid) (Gu et al., 2020). During production of high-value phenylpropanoid such as hydroxycinnamic acids, stilbenoids and flavonoids, formation of these undesired by-products represents a drain of precursors and goes at the expense of product titers and yields.

Fusel alcohols and acids are formed via the Ehrlich pathway for degradation of branched-chain, aromatic, and sulfur-containing amino acids (Ehrlich, 1907; Hazelwood et al., 2008). In the Ehrlich pathway, transamination of amino acids yields the corresponding 2-oxo acids, which are subsequently decarboxylated. The resulting aldehyde is then either oxidised or reduced by yeast aldehyde dehydrogenases and alcohol dehydrogenases to yield fusel acids and fusel alcohols, respectively (Hazelwood et al., 2008). The irreversible decarboxylation reaction in the Ehrlich pathway is catalysed by thiamine-pyrophosphate-dependent 2-oxo acid decarboxylases (2-OADC), which in *S. cerevisiae* are encoded by *PDC1*, *PDC5*, *PDC6* and *ARO10*. Pdc1, Pdc5 and Pdc6 show a preference for the linear-chain 2-oxo acids pyruvate, 2-oxobutyrate and 2-oxopentanoate (Romagnoli et al., 2012), while Aro10 shows no activity with linear chain 2-oxo acids, but does decarboxylate branched-chain and aromatic 2-oxo acids at high rates (Vuralhan et al., 2003, 2005; Romagnoli et al., 2012). Aro10 is a main contributor to 2-phenylethanol production by *S. cerevisiae* (Vuralhan et al., 2003; Romagnoli et al., 2012; Hassing et al., 2019), but Pdc5 also shows a distinct activity with phenylpyruvate (Romagnoli et al., 2012). Strains expressing heterologous pathways for flavonoid production from which *ARO10, PDC5* and *PDC6* were deleted, still exhibited residual 2-phenylethanol formation, indicating that Pdc1 still decarboxylated 2-phenylpyruvate at low rates (Koopman et al., 2012).

Deletion of the three pyruvate-decarboxylase genes *PDC1*, *PDC5*, *PDC6* renders *S. cerevisiae* unable to grow on glucose in batch cultures. During aerobic glucose-limited growth, Pdc^−^ strains are auxotrophic for C₂-compounds (ethanol or acetate) due to involvement of pyruvate decarboxylases in cytosolic acetyl-CoA synthesis (Flikweert et al., 1996, 1999). Although metabolic engineering strategies have been published to bypass this acetyl-CoA requirement (reviewed by Van Rossum et al. (van Rossum et al., 2016)) and to mitigate the glucose sensitivity of Pdc^−^ strains (Oud et al., 2012), deletion of all four 2-OADC genes in *S. cerevisiae* is not a straightforward strategy to eliminate formation of aromatic fusel alcohols and acids. Still, complete elimination of these by-products, without negative impacts on growth on glucose or a need to rewire central carbon metabolism, would be an attractive attribute of *S. cerevisiae* ‘chassis’ strains for production of phenylpropanoid.

The goal of the present study was to identify heterologous pyruvate decarboxylases that show activity with pyruvate but not with aromatic 2-oxo acids and to investigate whether they can functionally replace the native yeast enzymes and thereby prevent formation of aromatic Ehrlich-pathway products. To this end, a set of 11 2-oxo acid decarboxylases from yeasts (*K. lactis, K. marxianus* and *Y. lipolytica*) and bacteria (*Z. mobilis* and G*. diazotrophicus*) were expressed in a 2-OADC-deficient *S. cerevisiae* strain. Enzyme assays with cell extracts of the resulting strains were used to assess substrate specificities and affinities of each of the decarboxylases for pyruvate and phenylpyruvate. Subsequently, they were used to replace the native 2-OADC in a *S. cerevisiae* strain engineered for the production of the phenylpropanoid compound, coumaric acid. The impact of replacing the native yeast 2-OADC with the two best-performing heterologous pyruvate-decarboxylases was evaluated in aerobic, pH-controlled bioreactor cultures.

## 2 Material and methods

### 2.1 Strains and growth media

*S. cerevisiae* strains used in this study were derived from the CEN.PK lineage (Entian and Kötter, 2007; Salazar et al., 2017) (Table 1). *Escherichia coli* XL1 blue (Agilent Technologies, Santa Clara, CA) was used for plasmid propagation and storage. *S. cerevisia*e and *E. coli* strains were stored at −80 °C as described previously (Mans et al., 2015). Complex YP (yeast extract/peptone) medium was prepared and sterilized as described previously (Mans et al., 2015) and, when required, was supplemented with 200 mg L^−1^ hygromycin (InvivoGen, San Diego, CA). As a carbon source, YP medium was supplemented with either 20 g L^−1^ glucose (YPD) or a mixture of 2% (v/v) glycerol and 2% (v/v) ethanol (YPEG) (Mans et al., 2017). Chemically defined synthetic medium (SM) containing mineral salts, trace elements and vitamins was prepared and autoclaved as described by Verduyn et al. (Verduyn, 1991). SM was supplemented with either 20 g L^−1^ glucose (SMD), 2% (v/v) ethanol (SME) or a mixture of 2% (v/v) glycerol and 2% (v/v) ethanol (SMEG) (Mans et al., 2017). When required, 150 mg L^−1^ uracil (Sigma-Aldrich, St Louis, MO) (Pronk, 2002) was supplied to synthetic media. Lysogeny broth (LB) for growth of *E. coli* strains was prepared as described by Bertani et al. (Bertani, 1951) and supplied with 25 mg L^−1^ chloramphenicol (Sigma-Aldrich), 100 mg L^−1^ ampicillin (Sigma-Aldrich) or 50 mg L^−1^ kanamycin (Sigma-Aldrich) as required. Solid media were prepared by adding 20 g L^−1^ Bacto Agar prior to autoclaving.

**Table 1:** **Strains used in this study.** Abbreviations: *Sc Saccharomyces cerevisiae, Km Kluyveromyces marxianus*, *Kl Kluyveromyces lactis, Yl Yarrowia lipolytica*, *Gd Gluconacetobacter diazotrophicus, Zm Zymomonas mobilis, Rc Rhodobacter capsulatus, Pl Photorhabdus luminescens,* Co codon optimized*,* pr promoter, t terminator, pdc↓ *pdc5Δ pdc6Δ aro10Δ,* Pdc^−^*pdc1Δ pdc5Δ pdc6Δ aro10Δ,* 3ABP aromatic amino acid biosynthetic pathway, COUM coumaric acid, FBR feedback resistant, 2 *μ*m multicopy.

Strain	Description	Genotype	Reference
CEN.PK113-7D	Reference	MATa *MAL2-8c SUC2*	Entian and Kötter (2007)
CEN.PK711–7C	Pdc^−^*thi3Δ*	*MATa ura3-52 pdc1*Δ *pdc5Δ pdc6Δ aro10:: loxP-kan-loxP thi3::loxP-kan-loxP*	Vuralhan et al. (2005)
IMZ001	CEN.PK711–7C p426GPD	*MATa ura3-52 pdc1Δ pdc5Δ pdc6Δ aro10:: loxP-kan-loxP thi3::loxP-kan-loxP* p426GPD (*URA3*)	Vuralhan et al. (2005)
IMZ002	CEN.PK711–7C pUDE001	*MATa ura3-52 pdc1Δ pdc5Δ pdc6Δ aro10:: loxP-kan-loxP thi3::loxP-kan-loxP* pUDE001 (*URA3 TDH3pr-ScARO10-CYC1t*)	Vuralhan et al. (2005)
IMZ024	CEN.PK711–7C pUDE002	*MATa ura3-52 pdc1Δ pdc5Δ pdc6Δ aro10:: loxP-kan-loxP thi3::loxP-kan-loxP* pUDE002 (*URA3 TDH3pr-ScPDC5-CYC1t*)	Romagnoli et al. (2012)
IMZ031	CEN.PK711–7C pEXP214-PDC6.2	*MATa ura3-52 pdc1Δ pdc5Δ pdc6Δ aro10:: loxP-kan-loxP thi3::loxP-kan-loxP* pEXP214-PDC6.2 (*URA3 PGK1pr-ScPDC6-CYC1t*)	Romagnoli et al. (2012)
IMX1593	3ABP^FBR^	*MATa ura3-52 spr3Δ::Spcas9-*natNT2 *aro3Δ aro7Δ TDH3p-ARO4*^*K229L*^*-ARO4t ARO7pr::SeGPM1pr-ARO7*^*T226I*^*-TEF1t*	Hassing et al. (2019)
IMX1758	3ABP^FBR^ pdc↓pUDR406	*MATa ura3-52 spr3Δ::Spcas9-*natNT2 *aro3Δ aro7Δ TDH3p-ARO4*^*K229L*^*-ARO4t ARO7pr::SeGPM1pr-ARO7*^*T226I*^*-TEF1t pdc5Δ pcd6Δ aro10Δ pUR406* (*URA3,* gRNA-*PDC5/PDC6, ARO10*)	This study
IMX1789	3ABP^FBR^ pdc↓	*MATa ura3-52 spr3Δ::Spcas9-*natNT2 *aro3Δ aro7Δ TDH3p-ARO4*^*K229L*^*-ARO4t ARO7pr::SeGPM1pr-ARO7*^*T226I*^*-TEF1t pdc5Δ pcd6Δ aro10Δ*	This study
IMX2656	3ABP^FBR^ pdc↓ COUM	*MATa ura3-52 spr3Δ::Spcas9-*natNT2 *aro3Δ aro7Δ TDH3p-ARO4*^*K229L*^*-ARO4t ARO7pr::SeGPM1pr-ARO7*^*T226I*^*-TEF1t x3::SePDC1pr-*Co*PlstlA-ENO2t shrB TPI1pr-CoAtC4H-ADH1t PGI1or-CoAtCPR1-PGI1t shrC SkTDH3pr-CoRctal1-ADH1t pdc5Δ pcd6Δ aro10Δ*	This study
IMX2668	3ABP^FBR^ Pdc^−^ COUM	*MATa ura3-52 spr3Δ::Spcas9-*natNT2 *aro3Δ aro7Δ TDH3p-ARO4*^*K229L*^*-ARO4t ARO7pr::SeGPM1pr-ARO7*^*T226I*^*-TEF1t x3::SePDC1pr-*Co*PlstlA-ENO2t shrB TPI1pr-CoAtC4H-ADH1t PGI1or-CoAtCPR1-PGI1t shrC SkTDH3pr-CoRctal1-ADH1t pdc1Δ pdc5Δ pcd6Δ aro10Δ*	This study
IME418	CEN.PK711–7C pUDE833	MATa *MAL2-8c SUC2 ura3 pdc1Δ pdc5Δ pcd6Δ aro10Δ thi3Δ* pUDE833 (*URA3, TDH3pr-KlPDC5-CYC1t*)	This study
IME419	CEN.PK711–7C pUDE837	MATa *MAL2-8c SUC2 ura3 pdc1Δ pdc5Δ pcd6Δ aro10Δ thi3Δ* pUDE837 (*URA3 TDH3pr-YlPDC1-CYC1t*)	This study
IME420	CEN.PK711–7C pUDE838	MATa MAL2-8c SUC2 *ura3 pdc1Δ pdc5Δ pcd6Δ aro10Δ thi3Δ* pUDE838 (*URA3 TDH3pr-KmPDC1-CYC1t*)	This study
IME421	CEN.PK711–7C pUDE827	MATa *MAL2-8c SUC2 ura3 pdc1Δ pdc5Δ pcd6Δ aro10Δ thi3Δ* pUDE827 (*URA3 TDH3pr-CoZmpdc1-CYC1t*)	This study
IME422	CEN.PK711–7C pUDE829	*MATa MAL2-8c SUC2 ura3 pdc1Δ pdc5Δ pcd6Δ aro10Δ thi3Δ* pUDE829 (*URA3 TDH3pr-KmPDC5-CYC1t*)	This study
IME423	CEN.PK711–7C pUDE828	*MATa MAL2-8c SUC2 ura3 pdc1Δ pdc5Δ pcd6Δ aro10Δ thi3Δ* pUDE828 (*URA3 TDH3pr-KmARO10-CYC1t*)	This study
IME424	CEN.PK711–7C pUDE834	*MATa MAL2-8c SUC2 ura3 pdc1Δ pdc5Δ pcd6Δ aro10Δ thi3Δ* pUDE834 (*URA3 TDH3pr-KlARO10-CYC1t*)	This study
IME425	CEN.PK711–7C pUDE832	*MATa MAL2-8c SUC2 ura3 pdc1Δ pdc5Δ pcd6Δ aro10Δ thi3Δ* pUDE832 (*URA3 TDH3pr-CoGdpdc1.1-CYC1t*)	This study
IME474	CEN.PK711–7C pUDE881	*MATa MAL2-8c SUC2 ura3 pdc1Δ pdc5Δ pcd6Δ aro10Δ thi3Δ* pUDE881 (*URA3 TDH3pr-CoGdpdc1.2-CYC1t*)	This study
IME495	CEN.PK711–7C pUDE882	*MATa MAL2-8c SUC2 ura3 pdc1Δ pdc5Δ pcd6Δ aro10Δ thi3Δ* pUDE882 (*URA3 TDH3pr-CoGdpdc1.3-CYC1t*)	This study
IME615	CENPK711-7C pUDE1037	*MATa MAL2-8c SUC2 ura3 pdc1Δ pdc5Δ pcd6Δ aro10Δ thi3Δ* pUDE1037 (*URA3 TDH3pr-KlPDC1-CYC1t*)	This study
IME656	IMX2668 pGGKd017	*MATa ura3-52 spr3Δ::Spcas9-*natNT2 *aro3Δ aro7Δ TDH3p-ARO4*^*K229L*^*-ARO4t ARO7pr::SeGPM1pr-ARO7*^*T226I*^*-TEF1t x3::SePDC1pr-*Co*PlstlA-ENO2t shrB TPI1pr-CoAtC4H-ADH1t PGI1or-CoAtCPR1-PGI1t shrC SkTDH3pr-CoRctal1-ADH1t pdc1Δ pdc5Δ pcd6Δ aro10Δ pdc1Δ* pGGKd017 (*URA3*)	This study
IME658	IMX2668 pUDE827	*MATa ura3-52 spr3Δ::Spcas9-*natNT2 *aro3Δ aro7Δ TDH3p-ARO4*^*K229L*^*-ARO4t ARO7pr::SeGPM1pr-ARO7*^*T226I*^*-TEF1t x3::SePDC1pr-*Co*PlstlA-ENO2t shrB TPI1pr-CoAtC4H-ADH1t PGI1or-CoAtCPR1-PGI1t shrC SkTDH3pr-CoRctal1-ADH1t pdc1Δ pdc5Δ pcd6Δ aro10Δ* pUDE827 (*URA3 TDH3pr-CoZmpdc1-CYC1t*)	This study
IME659	IMX2668 pUDE838	*MATa ura3-52 spr3Δ::Spcas9-*natNT2 *aro3Δ aro7Δ TDH3p-ARO4*^*K229L*^*-ARO4t ARO7pr::SeGPM1pr-ARO7*^*T226I*^*-TEF1t x3::SePDC1pr-*Co*PlstlA-ENO2t shrB TPI1pr-CoAtC4H-ADH1t PGI1or-CoAtCPR1-PGI1t shrC SkTDH3pr-CoRctal1-ADH1t pdc1Δ pdc5Δ pcd6Δ aro10Δ* pUDE838 (*URA3 TDH3pr-KmPDC1-CYC1t*)	This study
IME660	IMX2668 pUDE837	*MATa ura3-52 spr3Δ::Spcas9-*natNT2 *aro3Δ aro7Δ TDH3p-ARO4*^*K229L*^*-ARO4t ARO7pr::SeGPM1pr-ARO7*^*T226I*^*-TEF1t x3::SePDC1pr-*Co*PlstlA-ENO2t shrB TPI1pr-CoAtC4H-ADH1t PGI1or-CoAtCPR1-PGI1t shrC SkTDH3pr-CoRctal1-ADH1t pdc1Δ pdc5Δ pcd6Δ aro10Δ* pUDE837 (*URA3 TDH3pr-YlPDC1-CYC1t*)	This study
IME661	IMX2668 pUDE1037	*MATa ura3-52 spr3Δ::Spcas9-*natNT2 *aro3Δ aro7Δ TDH3p-ARO4*^*K229L*^*-ARO4t ARO7pr::SeGPM1pr-ARO7*^*T226I*^*-TEF1t x3::SePDC1pr-*Co*PlstlA-ENO2t shrB TPI1pr-CoAtC4H-ADH1t PGI1or-CoAtCPR1-PGI1t shrC SkTDH3pr-CoRctal1-ADH1t pdc1Δ pdc5Δ pcd6Δ aro10Δ* pUDE1037 (*URA3 TDH3pr-Klpdc1-CYC1t*)	This study
IME662	IMX2668 pUDE881	*MATa ura3-52 spr3Δ::Spcas9-*natNT2 *aro3Δ aro7Δ TDH3p-ARO4*^*K229L*^*-ARO4t ARO7pr::SeGPM1pr-ARO7*^*T226I*^*-TEF1t x3::SePDC1pr-*Co*PlstlA-ENO2t shrB TPI1pr-CoAtC4H-ADH1t PGI1or-CoAtCPR1-PGI1t shrC SkTDH3pr-CoRctal1-ADH1t pdc1Δ pdc5Δ pcd6Δ aro10Δ* pUDE881 (*URA3 TDH3pr-CoGdpdc1.2-CYC1t*)	This study
IME663	IMX2668 pUDE882	*MATa ura3-52 spr3Δ::Spcas9-*natNT2 *aro3Δ aro7Δ TDH3p-ARO4*^*K229L*^*-ARO4t ARO7pr::SeGPM1pr-ARO7*^*T226I*^*-TEF1t x3::SePDC1pr-*Co*PlstlA-ENO2t shrB TPI1pr-CoAtC4H-ADH1t PGI1or-CoAtCPR1-PGI1t shrC SkTDH3pr-CoRctal1-ADH1t pdc1Δ pdc5Δ pcd6Δ aro10Δ* pUDE882 (*URA3 TDH3pr-CoGdpdc1.3-CYC1t*)	This study
IME667	CENPK711-7C pUDE1099	*MATa MAL2-8c SUC2 ura3 pdc1Δ pdc5Δ pcd6Δ aro10Δ thi3Δ* pUDE1099 (*URA3 TDH3pr-ScPDC1-CYC1t*)	This study
IME668	CENPK711-7C pUDE1101	*MATa MAL2-8c SUC2 ura3 pdc1Δ pdc5Δ pcd6Δ aro10Δ thi3Δ* pUDE1101 (*URA3 TDH3pr-ScPDC1-CYC1t ENO2pr-ScTHI3-GPM1t*)	This study
IME677	IMX2668 pUDE1099	*MATa ura3-52 spr3Δ::Spcas9-*natNT2 *aro3Δ aro7Δ TDH3p-ARO4*^*K229L*^*-ARO4t ARO7pr::SeGPM1pr-ARO7*^*T226I*^*-TEF1t x3::SePDC1pr-*Co*PlstlA-ENO2t shrB TPI1pr-CoAtC4H-ADH1t PGI1or-CoAtCPR1-PGI1t shrC SkTDH3pr-CoRctal1-ADH1t pdc1Δ pdc5Δ pcd6Δ aro10Δ* pUDE1099 (*URA3 TDH3pr-ScPDC1-CYC1t*)	This study

### 2.2 Molecular biology techniques

DNA templates for cloning were amplified with Phusion high-fidelity polymerase (Thermo Fisher Scientific, Landsmeer, Netherlands) according to manufacturer's protocol, with the exception that a primer concentration of 200 nM and 0.04 U μL^−1^ of polymerase were used. The YeaStar genomic DNA kit (Zymo Research, Irvine, CA) was used to isolate genomic DNA as template for PCR amplification. The Zymoclean kit (Zymo Research) was used to purify PCR products by gel purification according to manufacturer's recommendations using milliQ water as eluent. Alternatively, PCR products were first incubated for 1 h with DpnI FastDigest enzyme (Thermo Fisher Scientific) to digest template DNA and subsequently purified using the GenElute™ PCR clean-Up Kit (Sigma-Aldrich). Diagnostic PCR was performed with DreamTaq PCR mastermix (Thermo Fisher Scientific) and with oligonucleotide primers shown in Table S1 The GenElute plasmid miniprep kit (Sigma-Aldrich) was used to isolate plasmids from *E. coli*.

### 2.3 Construction of plasmids and expression cassettes

Plasmids used and constructed in this study are shown in Table 2. Constructed plasmids were transformed to *E. coli* (XL1-Blue) cells according to the supplier's recommendations and grown under selective conditions.

**Table 2:** **Plasmids used in this study**. Abbreviations: *Sc Saccharomyces cerevisiae, Km Kluyveromyces marxianus, Kl Kluyveromyces lactis, Yl Yarrowia lipolytica, Gd Gluconacetobacter diazotrophicus, Zm Zymomonas mobilis, Rc Rhodobacter capsulatus; Pl Photorhabdus luminescens*, Co codon optimized, pr promoter, t terminator, DO dropout.

Part Plasmids			
Name	Description	Part Type	Source
JA_NM_1 Sc_CoPlstlA	*camR CoPlstlA*	3	This study
pYTK001	*camR GFP* entry vector	Insert	Lee et al. (2015)
pYTK002	*camR* ConLS connector	1	Lee et al. (2015)
pYTK003	*camR* ConLS′ connector	1	Lee et al. (2015)
pYTK008	*camR* ConL1 connector	1	Lee et al. (2015)
pYTK047	*camR GFP* DO	234r	Lee et al. (2015)
pYTK055	*camR ENO2t*	5	Lee et al. (2015)
pYTK067	*camR* ConR1 connector	5	Lee et al. (2015)
pYTK072	*camR* ConRE connector	5	Lee et al. (2015)
pYTK073	*camR* ConRE’ connector	5	Lee et al. (2015)
pYTK074	*camR URA3*	6	Lee et al. (2015)
pYTK082	camR 2 *μ*m	7	Lee et al. (2015)
pYTK084	*camR kanR-ColE1*	8	Lee et al. (2015)
pUD565	*camR GFP* entry vector	Insert	Boonekamp et al. (2018)
pGGKp025	*camR PDC1pr*	2	Hassing et al. (2019)
pGGKp027	*camR FBA1pr*	2	This study
pGGKp028	*camR ENO2pr*	2	Hassing et al. (2019)
pGGKp035	*camR TDH3pr*	2	Hassing et al. (2019)
pGGKp037	*camR ADH1t*	4	Hassing et al. (2019)
pGGKp039	*camR TEF1t*	4	Hassing et al. (2019)
pGGKp045	*camR PDC1t*	4	Hassing et al. (2019)
pGGKp048	*camR GPM1t*	4	Hassing et al. (2019)
pGGKp063	*camR SkTDH3pr*	2	Hassing et al. (2019)
pGGKp074	*camR SePDC1pr*	2	Hassing et al. (2019)
pGGKp182	*camR CYC1t*	4	This study
pGGKp183	*camR KmARO10*	3	This study
pGGKp184	*camR KmPDC1*	3	This study
pGGKp185	*camR* putative *KmPDC5*	3	This study
pGGKp211	*camR CoZmpdc1*	3	This study
pGGKp212	*camR CoGdpdc1.1*	3	This study
pGGKp213	*camR KlPDC5*	3	This study
pGGKp214	*camR KlARO10*	3	This study
pGGKp254	*camR CoGdpdc1.2*	3	This study
pGGKp255	*camR CoGdpdc1.3*	3	This study
pGGKp314	*camR KlPDC1*	3	This study
pGGKp315	*camR ScTHI3*	3	This study
pGGKp327	*CoRctal1*	3	This study
pGGKp337	*ScPDC1*	3	This study
**Expression cassettes**			
**Plasmid**	**Genotype**	**Parts used**	**Source**
pGGKd017	2 *μ*m *ampR-ColE1 URA3 GFP* DO	pYTK002, pYTK047, pYTK72, pYTK074, pYTK082, pYTK083	Wronska et al. (2020)
pGGKd071	2 *μ*m *ampR-ColE1 conLS conR1 URA3 GFP* DO	pYTK002, pYTK047, pYTK67, pYTK074, pYTK082, pYTK083	This study
pGGKd072	2 *μ*m *ampR-ColE1 conL1 conRS URA3 GFP* DO	pYTK003, pYTK047, pYTK72, pYTK074, pYTK082, pYTK083	This study
pGGKd073	2 *μ*m *kanR-ColE1 conLS′ conRS′ URA3 GFP* DO	pYTK008, pYTK047, YTK073, pYTK074, pYTK082, pYTK084	This study
pUDE827	2 *μ*m *ampR URA3 TDH3pr-CoZmpdc1-CYC1t*	pGGKd017, pGGKp035, pGGKp182 pGGKp211	This study
pUDE828	2 *μ*m *ampR URA3 TDH3pr-KmARO10-CYC1t*	pGGKd017, pGGKp035, pGGKp182 pGGKp183	This study
pUDE829	2 *μ*m *ampR URA3 TDH3pr-KmPDC5-CYC1t*	pGGKd017, pGGKp035, pGGKp182 pGGKp185	This study
pUDE832	2 *μ*m *ampR URA3 TDH3pr-CoGdpdc1.1-CYC1t*	pGGKd017, pGGKp035, pGGKp182 pGGKp212	This study
pUDE833	2 *μ*m *ampR URA3 TDH3pr-KlPDC5-CYC1t*	pGGKd017, pGGKp035, pGGKp182 pGGKp213	This study
pUDE834	2 *μ*m *ampR URA3 TDH3pr-KlARO10-CYC1t*	pGGKd017, pGGKp035, pGGKp182 pGGKp214	This study
pUDE837	2 *μ*m *ampR URA3 TDH3pr-YlPDC1-CYC1t*	Gibson assembly, pGGKd017, pGGKd035, pGGKp182	This study
pUDE838	2 *μ*m *ampR URA3 TDH3pr-KmPDC1-CYC1t*	pGGKd017, pGGKp035, pGGKp182 pGGKp184	This study
pUDE881	2 *μ*m *ampR URA3 TDH3pr -CoGdpdc1.2-CYC1t*	pGGKd017, pGGKp035, pGGKp254, pGGKp182	This study
pUDE882	2 *μ*m *ampR URA3 TDH3pr-CoGdpdc1.3-CYC1t*	pGGKd017, pGGKp035, pGGKp255, pGGKp182	This study
pUDE1019	2 *μ*m *ampR URA3 SePDC1pr-CoPlstlA-ENO2t*	pGGKd017, pGGKp074, pYTK055, JA_NM 1_ Sc*_coPlstIA*	This study
pUDE1037	2 *μ*m *ampR URA3 TDH3p-KlPDC1-CYC1t*	pGGKd017, pGGKp035, pGGKp314, pGGKp182	This study
pUDE1049	2 *μ*m *ampR URA3 ENO2pr-ScTHI3-GPM1t*	pGGkd072, pGGKp028, pGGKp315, pGGKp048	This study
pUDE1088	2 *μ*m *ampR URA3 SkTDH3pr-CoRctal1-CYC1t*	pGGkd017, pGGKp037, pGGKp063, pGGKp327	This study
pUDE1099	2 *μ*m *ampR URA3 TDH3pr-ScPDC1-CYC1t*	pGGKd017, pGGKp035, pGGKp182, pGGKp337	This study
pUDE1100	2 *μ*m *ampR URA3 TDH3pr-ScPDC1-CYC1t*	pGGKd071, pGGKp035, pGGKp182, pGGKp337	This study
pUDE1101	2 *μ*m *kanR URA3 TDH3pr-ScPDC1-CYC1t, ENO2pr-ScTHI3-GPM1t*	pGGd073, pUDE1049, pUDE1100	This study
**Cas9 Plasmids**			
**Name**	**Relevant Genotype**	**Primer(s) used for gRNA**	**Source**
pROS10	2 *μ*m *bla URA3* gRNA-*CAN1*.Y gRNA-*ADE2.Y*	N.A.	Mans et al. (2015)
pROS12	2 *μ*m *bla* hphNT1 gRNA-*CAN1*.Y gRNA*-ADE2.Y*	N.A.	Mans et al. (2015)
pUDR406	2 *μ*m *bla URA3* gRNA-*PDC5/PDC6* gRNA-*ARO10*	7246 & 13614	This Work
pUDR470	2 *μ*m *bla* hphNT1 gRNA-*PDC1* (2x)	6178	This Work
pUDR599	2 *μ*m *bla* hphNT1 gRNA-*X3* (2x)	15832	Hassing et al. (2019)
**Miscellaneous Plasmids**			
**Name**	**Relevant Genotype**		**Source**
pUDE172	Centromeric plasmid, *URA3 TDH3pr-AtPAL1-CYC1t TPIp-CoAtC4H-ADHt PGIpr-CoAtCPR1-PGIt*		Koopman et al. (2012)
pUDI069	Integration plasmid, *TRP1 TDH3pr-CoRctal1-CYC1t*		Koopman et al. (2012)
pE_MGV14	2 *μ*m *TDH3pr* natNT2 *bla HXT7p,-CoPlstlA-CYC1t*		(Gottardi et al., 2017b)

Plasmids containing gRNAs for Cas9-mediated genome editing were constructed as described by Mans et al. (2015). The resulting gRNA plasmids pUDR406 (gRNA-*PDC5/PDC6* and gRNA-*ARO10*), pUDR470 (gRNA-*PDC1* 2x) and pUDR599 (gRNA-X3 2x) (Hassing et al., 2019) were used to target *PDC5, PDC6*, *PDC1*, *ARO10* and the *X*3-locus (Mikkelsen et al., 2012), respectively.

The expression cassettes used in this study were constructed using the Yeast Toolkit (Lee et al., 2015). In brief, promoter, gene and terminator fragments (parts) are amplified with part type specific overhangs containing restriction sites (BsmBI and BsaI). Using Golden Gate assembly with the corresponding restriction enzyme, BsmBI, the individual parts are initially assembled in an universal entry vector, resulting in a part plasmid. Next, a promoter, gene and terminator part plasmid are assembled into an expression cassette using BsaI-mediated golden gate assembly, resulting in an expression cassette containing a transcriptional unit.

Initially, DNA fragments carrying the *CYC1* terminator (*CYC1*t) and *FBA1* promoter (*FBA1*pr) fragments were amplified from genomic DNA from *S. cerevisiae* CEN.PK113-7D with oligonucleotide primers 14039/14040 and 9419/9420 adding terminator (ATCC and CAGC) or promoter (AACG and ATAC) part type specific overhangs (Table S1) (Lee et al., 2015). Open reading frames of genes from *S. cerevisiae* CEN.PK113-7D (Entian and Kötter, 2007), *Kluyveromyces marxianus* NRBC 1777 (NITE Biological Resource Center, Japan) (Inokuma et al., 2015) or *Kluyveromyces lactis* CBS 2359 (Juergens et al., 2018) were amplified from genomic DNA using primers with gene-part type specific overhangs (TATG and GGAT) (Table S1). Primers ASR_A023F/ASR_A023Rcorr, 13940/13941 and 16851/16852 and 17630/17631 were used to amplify *KmPDC1, KlARO10, ScPDC1* and *ScTHI3* respectively. The ORFs of *KlPDC5*, *KlPDC1, KmPDC5 and KmARO10* were amplified in several fragments to remove internal BsaI and BsmBI sites from the coding sequence (Hassing et al., 2019) using primer pairs 13932/13933 and 13934/13935 for *KlPDC5,* 13939/14138, 14137/13938 and 13937/13936 for *KlPDC1,* ASR_A024F/ASR_A024MR and ASR_A024MF/AR_A024Rcorr for *KmPDC5* and ASR_A022F/ASR_A022MR and ASR_A022MF/ASR_A022Rcorr for *KmARO10. CoRctal1* was amplified using pUDI069 (Koopman et al., 2012) as template with primers 17825/17826. Correct removal of the internal BsaI/BsmBI sites of *KmPDC5, KmARO10, KlPDC5* and *KlPDC1* was confirmed by Sanger sequencing (BaseClear, Leiden, Netherlands). A codon-optimized, based on yeast glycolytic codon usage (Wiedemann and Boles, 2008), open reading frame of the phenylalanine ammonia lyase gene from *Photorhabdus luminescens, CoPlstLA,* was amplified from plasmid MGV14 (*CoPlstLA*) (Gottardi et al., 2017) with primers ASR_N009F/ASR_N009R. Codon optimisation of the *Gdpdc1* and *Zmpdc1* coding regions was performed using the Jcat Codon Adaptation Tool (Grote et al., 2005). The codon regions were custom-synthesized by Invitrogen GeneArt (Thermo Fisher Scientific) service. The sequence of *Zmpdc1* was derived from the annotated genome of strain *Zymomonas mobilis* subsp. *mobilis* ATCC 10988 (Bioproject accession number PRJNA30987) (Pappas et al., 2011)*.* Since three different sequences of *Gdpdc1* have been reported for *Gluconacetobacter diazotrophicus* strain ATCC 49037, codon-optimized coding sequences for *Gdpdc1.1* (van Zyl et al., 2014)*, Gdpdc1.2* (Bertalan et al., 2009) and *Gdpdc1.3* (Giongo et al., 2010) were separately synthesized. Coding sequences were flanked upstream and downstream with the gene specific Yeast Toolkit flanks ‘AAGCATCGTCTCATCGGTCTCAT’ and ‘TTATGCCGTCTCAGGTCTCAGGAT’ respectively (Lee et al., 2015).

The amplified and synthesized fragments of *CYC1t, KmARO10, KmPDC1, KmPDC5* and *CoPlstLA* were cloned into entry vector pYTK001 (Lee et al., 2015), via BsmBI Golden Gate assembly, obtaining part plasmids pGGKp182 (*CYC1t),* pGGKp183 (*KmARO10*), pGGKp184 (*KmPDC1*), pGGKp185 (*KmPDC5*) and JA_NM 1_Sc_coPlstlA (*CoPlstLA*). *FBA1pr, Zmpdc1, Gdpdc1.1-3, KlPDC1*, *KlARO10, ScPDC1, KlPDC5, CoRctal1* and *ScTHI3* were also assembled via BsmBI Golden Gate assembly but into entry vector pUD565 (Boonekamp et al., 2018), resulting in part plasmids pGGKp027 (*FBA1pr*), pGGKp211 (*Zmpdc1*)*,* pGGKp212 (*Gdpdc1.1*), pGGKp213 (*KlPDC5*), pGGKp214 (*KlARO10*), pGGKp254 (*Gdpdc1.2*), pGGKp255 (*Gdpdc1.3*), pGGKp314 (*KlPDC1*), pGGKp315 (*ScTHI3*), pGGKp327 (*CoRctal1*) and pGGKp337 (*ScPDC1*). Part plasmids were confirmed by colony PCR using primers 2012 and 2397 for the pUD565 entry vector and with primers 14036 and 14977 for YTK001 entry vector.

The GFP dropout plasmid pGGKd017 (*URA3*) (Wronska et al., 2020) was used as backbone to construct expression cassettes expressing a single 2-oxo acid decarboxylase. As example, the Golden Gate assembly of pGGkp035 (*TDH3pr*), pGGkp182 (*CYC1t*) and pGGKp211 (*Zmpdc1*) using pGGKd017 as a backbone resulted in the construction of pUDE827 (*URA3, TDH3pr-Zmpdc1-CYC1t*). A full overview of all part plasmids that were used to construct the expression cassettes is presented in Table 2. Correct construction was verified by diagnostic PCR and restriction analysis.

Additionally, a multi-expression cassette plasmid expressing *ScPDC1* and *ScTHI3* was constructed*.* For this purpose, three additional GFP dropout plasmids were first constructed. The part plasmids pYTK002 and pYTK067 (ConLS and ConR1 connectors), pYTK047 (GFP dropout), pYTK074 (*URA3*), pYTK082 (2 *μ*m,) with pYTK083 (ColE1 *bla*) were assembled via BsmBI Golden Gate assembly (Lee et al., 2015) resulting in pGGKd071 (multigene cassette #1). Additionally pYTK003 and pYTK072 (ConL1 and ConRE connectors), pYTK047, pYTK074, pYTK082 were assembled via BsmBI Golden Gate assembly (Lee et al., 2015) resulting in pGGKd072 (multigene cassette #2). Finally, pGGKd073, a GFP multigene dropout plasmid, was constructed by assembling pYTK008 and pYTK073 (ConLS′ and ConRE’ connectors), pYTK047, pYTK074, pYTK082 with pYTK084 (ColE1 *nptII*).

After this, using BsaI mediated golden gate assembly, pGGkd072 (multigene cassette #2), pGGKp028 (*ENO2pr*), pGGKp315 (*ScTHI3*) and pGGKp048 (*GPM1t*) were assembled resulting in pUDE1049 (*ScTHI3,* multigene cassette #2). Next, pGGkd071 (multigene cassette #1), pGGKp035 (*TDH3pr*), pGGKp337 (*ScPDC1*) and pGGKp182 (*CYC1t*) were assembled resulting in pUDE1100 (*ScPDC1* multigene cassette #1). Finally, pGGKd073 (multigene dropout), pUDE1049 and pUDE1100 were assembled using a BsmBI golden gate assembly into pUDE1101 (*ScPDC1, ScTHI3*) (Table 2). Final plasmid confirmation was done by restriction analysis.

The expression cassette bearing *YlPDC1* was assembled using Gibson assembly. The gene *YlPDC1* (YALI0D10131g, Genome Resources for Yeast Chromosomes database (https://gryc.inra.fr)) was PCR amplified from genomic DNA of *Y. lipolytica* W29 (Magnan et al., 2016; Wronska et al., 2020) using primers 14187/14188. The *TDH3pr* and *CYC1t* were amplified from pGGKp035 and pGGKp182 using primers 14185/14186 and 14189/14190, respectively. The linear pGGKd017 backbone was amplified using primers 14183/14184. The plasmid pUDE837 (*YlPDC1*) was constructed using Gibson assembly of the promoter, gene and terminator fragments. Correct construction of pUDE837 was confirmed by restriction analysis.

### 2.4 Strain construction

The thiamine-pyrophosphate-dependent-decarboxylase-negative strain *S. cerevisiae* CENPK711-7C (*ura3Δ pdc1Δ pdc5Δ pdc6Δ aro10Δ thi3Δ*) (Vuralhan et al., 2005) was transformed with 2-oxo acid decarboxylase-expressing episomal (2 μm) plasmids resulting in strains (pUDE833 (*KlPDC5↑*)), IME419 (pUDE837 (*YlPDC1↑*)), IME420 (pUDE838 (*KmPDC1↑*)), IME421 (pUDE827 (*Zmpdc1↑*)), IME422 (pUDE829 (*KmPDC5↑*)), IME423 (pUDE828 (*KmARO10↑*)), IME424 (pUDE834 (*KlARO10↑*)), IME425 (pUDE832 (*Gdpdc1.1↑*)), IME474 (pUDE881 (*Gdpdc1.2↑*)), IME495 (pUDE882 (*Gdpdc1.3↑*)), IME615 (pUDE1037 (*KlPDC1↑*)), IME667 (pUDE1099 (*ScPDC1↑*)) and IME668 (pUDE1101 (*ScPDC1↑ ScTHI3↑*).

*S. cerevisiae* IMX1593 (*ura3Δ Spcas9 aro3Δ aro7Δ ARO4*^*K229L*^*↑ ARO7*^*T226I*^*↑*) (Hassing et al., 2019) was used as starting point for construction of a coumaric acid producing strain. Transformation with pUDR406 (gRNA-*PDC5/PDC6, ARO10*) and repair fragments consisting of oligonucleotides 7247/7248 for *ARO10,* 7717/7718 for *PDC5* and 7935/7936 for *PDC6*, yielded strain IMX1758. After curing of pUDR406 (Mans et al., 2015) strain IMX1789 was obtained, into which expression cassettes for coumaric acid biosynthesis were integrated. Cassettes for expression of *CoRctal1, CoPlstlA* and *CoAtC4H/CoAtCPR1* were amplified using primer pairs 12044/18181, 12040/18183 and 4640/18180 and plasmids pUDE1088, pUDE1019 and pUDE172 (Koopman et al., 2012), respectively, as templates. Strain IMX1789 (*pdc5Δ pdc6Δ aro10Δ*) was co-transformed with pUDR599 (gRNA-X3) and the three expression cassettes *CoRctal1, CoPlstlA* and *CoAtC4H/CoAtCPR1* containing homologous flanks to the X3 locus or a short homologous sequence (shr) (Kuijpers et al., 2013) to allow homologous recombination of the flanks and integration into the edited X3 locus (Mikkelsen et al., 2012) resulting in strain IMX2656 after curing pUDR599. In the final step, *PDC1* was deleted by co-transforming strain IMX2656 (coumaric acid producing *pdc5Δ pdc6Δ aro10Δ*) with pUDR470 (gRNA-*PDC1*) and a repair fragment consisting of annealed oligonucleotides 7719 and 7720, resulting in strain IMX2668 after curing the gRNA plasmid.

The resulting strain IMX2668 (coumaric acid producing, *pdc1Δ pdc5Δ pdc6Δ aro10Δ*) was transformed with episomal plasmids expressing an individual 2-oxo-acid decarboxylase or with pGGKd017, an empty backbone plasmid, as negative control. This yielded strains IME656 (pGGKd017 (*URA3,* empty plasmid)), IME658 (pUDE827 (*Zmpdc1↑*))*,* IME659 (pUDE838 (*KmPDC1↑*))*,* IME660 (pUDE837 (*YlPDC1↑*))*,* IME661 (pUDE1037 (*KlPDC1↑*))*,* IME662 (pUDE881 (*Gdpdc1.2↑*))*,* IME663 (pUDE882 (*Gdpdc1.3↑*)) and IME677 (pUDE1099 (*ScPDC1↑*)).

### 2.5 Growth studies

Shake-flask cultures were grown in 500 mL shake flasks containing 100 mL medium and incubated at 30 °C in an Innova incubator shaker (New Brunswick Scientific, Edison, NJ). Precultures on SMEG were inoculated from frozen stock cultures. These precultures were used to inoculate shake flasks containing SMEG and SMD, at an initial OD_660_ of 0.2. Independent duplicate cultures were grown for each combination of yeast strain and medium composition. Specific growth rates were calculated from a minimum number of six data points collected during exponential growth and covering 3–4 doublings of OD_660_. Ehrlich pathway products were quantified in supernatant samples of triplicate stationary phase (72 h) shake-flask cultures.

Aerobic bioreactor batch cultures on SMD supplemented with 0.2 g L^−1^ antifoam C (Sigma-Aldrich) were grown in 2L bioreactors (Applikon, Delft, Netherlands) with a working volume of 1.0 L. Oxygen was supplied by continuously sparging the culture with pressurized air at 0.5 L min ^−1^. Exponentially growing shake-flask cultures on SMD were used to inoculate the bioreactors at an initial biomass concentration of around 0.1 g L^−1^. Cultures were grown at 30 °C and stirred at 800 rpm with a Rushton impeller. The culture pH was maintained at 5.0 by automated addition of 2 M KOH or 2 M H_2_SO_4_. Optical density at 660 nm was measured with a Jenway 7200 spectrophotometer (Jenway, Staffordshire, United Kingdom). Biomass dry weight was measured as described previously (Postma et al., 1989a). Off-gas from the bioreactors was cooled using a condenser and dried using a Permapure MD-110-48P-4 dryer (Permapure, Lakewood, NJ). CO_2_ and O_2_ concentrations in the off-gas were measured with a NGA 2000 Rosemount gas analyser (Rosemount, Analytical, Irvine, CA).

Concentrations of glucose, ethanol and extracellular organic acids in culture supernatants were measured by high performance liquid chromatography (HPLC) as described before (Hassing et al., 2019). The Ehrlich pathway metabolites 2-phenylethanol, *p*-hydroxyphenylethanol, phenylacetate, phenylpyruvate, coumaric acid and cinnamic acid were also measured by HPLC as described before (Hassing et al., 2019). Aromatic compounds were detected by a diode-array multiple-wavelength detector (Agilent G1315C), at wavelengths of 200 nm for phenylacetate, 210 nm for phenylpyruvate, 214 nm for 2-phenylethanol and *p*-hydroxyphenylethanol, 270 nm for cinnamic acid and 280 nm for coumaric acid.

### 2.6 Enzyme-activity assays in cell extracts

Cell extracts of *S. cerevisiae* strains were prepared from late exponential phase (OD_660_ of approximately 8) shake-flask cultures grown on SMEG or SME medium. After 10 min centrifugation at 4696×*g*, cell pellets were washed twice with 20 mL 10 mM potassium phosphate buffer (pH 7.5) containing 2 mM EDTA, resuspended in 4 mL buffer and stored at −20 °C. Prior to the enzyme assays, biomass samples were thawed, resuspended and washed with 100 mM potassium phosphate buffer (pH 7.5) containing 2 mM MgCl_2_ and 2 mM dithiothreitol. When cell extracts were prepared for experiments to estimate kinetic parameters, which took several hours, complete(TM), Mini Protease Inhibitor Co. (Sigma-Aldrich) was added as protease inhibitor according to manufacturer's recommendations. Cell extracts were prepared by sonication with 0.7 mm diameter glass beads using a MSE sonicator (150-W output, 7-nm peak-to-peak amplitude) at 0 °C. After four bursts of 30 s with 30 s cooling intervals, debris was removed by centrifugation using a Sorvall SS34-rotor (Thermo Fisher Scientific) for 20 min at 47.000×*g* operated at 4 °C. The clear supernatants were used as cell extracts and kept on ice during experiments.

Pyruvate-decarboxylase activity in cell extracts was measured as described previously (Postma et al., 1989b). Phenylpyruvate-decarboxylase activity was assayed essentially as described before (Vuralhan et al., 2003) but with 5 mM instead of 2 mM phenylpyruvate. *K*_*m*_ values for pyruvate were obtained by measuring pyruvate-decarboxylase activities at concentrations ranging from 0.1 mM to 50 mM, followed by nonlinear regression of the obtained results with GraphPad Prism (version 9.02, GraphPad Software, San Diego, CA). Datasets were fitted with Michaelis-Menten as well as allosteric sigmoidal kinetics.

### 2.7 Protein homology and phylogenetic tree

The amino acid sequences (Supplemental Dataset S.1) of the 2-oxo acid decarboxylases used in this study were aligned using Clustal Ώ (Sievers et al., 2011). A heat map displaying sequence similarity was generated using GraphPad Prism. A phylogenetic tree of the aligned protein sequences was constructed with SeaView5 (Gouy et al., 2010) applying the LG model (Le and Gascuel, 2008) with default parameter settings using 100 Bootstrap replicates as support level for internal branches.

## 3 Results

### 3.1 Selection of heterologous pyruvate-decarboxylases with a potentially narrow substrate specificity

Heterologous pyruvate-decarboxylases with a potentially better substrate selectivity for pyruvate were selected based on three criteria: i) homology with *S. cerevisiae* pyruvate decarboxylases, ii) a demonstrated or proposed role in pyruvate decarboxylation, iii) absence of evidence for activity with aromatic 2-oxo acids. A resulting set of 11 decarboxylases was selected comprised of *Kl*Pdc1 (Dujon et al., 2004)*, Kl*Pdc5 (Choo et al., 2018) and *Kl*Aro10 (Dujon et al., 2004) from *K. lactis, Km*Pdc1*, Km*Pdc5 and *Km*Aro10 from *K. marxianus* (Lertwattanasakul et al.*, 2015*)*, Yl*Pdc1 from *Y. lipolytica* (Dujon et al., 2004) and four bacterial pyruvate decarboxylases: *Zm*Pdc1 *from Z. mobilis* (Neale et al., 1988) and *Gd*Pdc1.1 (van Zyl et al., 2014)*, Gd*Pdc1.2 (Bertalan et al., 2009) and *Gd*Pdc1.3 (Giongo et al., 2010) from *G. diazotrophicus* (Table 3)*.* In subsequent experiments, these heterologous enzymes were compared with the native *S. cerevisiae* 2-oxo acid decarboxylases *Sc*Pdc1*, Sc*Pdc5*, Sc*Pdc6 and *Sc*Aro10.

**Table 3:** **Heterologous 2-oxo-acid decarboxylase (2-OADC) genes investigated in this study.** Published information on activity with pyruvate and with the aromatic 2-oxo acids phenylpyruvate (PPY) and *p-*hydroxyphenylpyruvate (*p*OHPPY) is presented. The right-hand column indicates for which of these genes the coding sequences were codon-optimized (Co) for expression in *S. cerevisiae* in the present study.

Organism	Gene	Activity with pyruvate	Activity with PPY or *p*OHPPY	Co
*K. marxianus*	*KmPDC1*	Yes (Choo et al., 2018)	Unknown	No
	*KmPDC5*	No (Choo et al., 2018)	Unknown	No
	*KmARO10*	Unknown	Unknown	No
*K. lactis*	*KlPDC1*	Yes (Bianchi et al., 1996)	Unknown	No
	*KlPDC5*	Unknown	Unknown	No
	*KlARO10*	Unknown	Suggested (Uzunov et al., 2014)	No
*Y. lipolytica*	*YlPDC1*	Suggested (Beopoulos et al., 2009)	Unknown	No
*Z. mobilis*	*Zmpdc1*	Yes (Bringer-Meyer et al., 1986)	not PPY (Siegert et al., 2005) low *p*OHPPY (Siegert et al., 2005)	Yes
*G. diazotrophicus*	*Gdpdc1.1*	Yes (van Zyl et al., 2014)	Not *p*OHPPY (van Zyl et al., 2014)	Yes
	*Gdpdc1.2*	Unknown	Unknown	Yes
	*Gdpdc1.3*	Unknown	Unknown	Yes

**Fig. 2:**
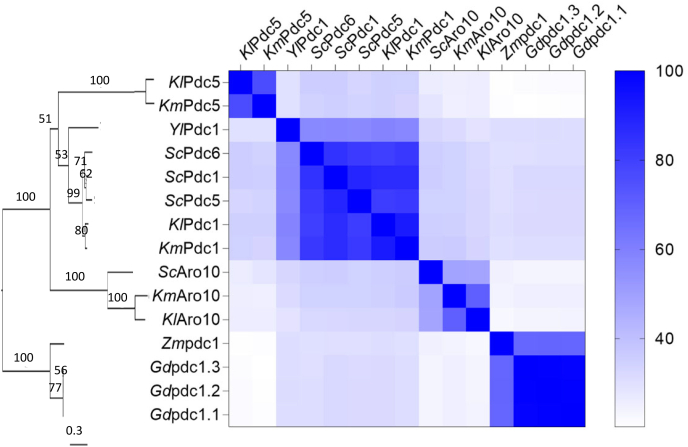
**Phylogenetic tree and protein similarity of the different 2-oxo acid decarboxylase candidates screened in this study.** The phylogentic tree was constructed as described in section 2.7 and the number of amino acid substitutions per site are represented by the scale bar. Protein similarity is represented in a heatmap. The greater the intensity of blue, the higher the amino acid homology between two candidates.

A phylogenetic tree of the amino-acid sequences of the selected 2-OADCs generated by multiple-sequence alignment using Clustal Ώ (Sievers et al., 2011) showed a clear segregation of the eukaryotic (yeast) and bacterial sequences (Fig. 2). As anticipated, sequences of the *S. cerevisiae* pyruvate decarboxylases Pdc1 and Pdc5 clustered with those of the *K. marxianus* and *K. lactis* Pdc1 orthologs. Interestingly, *Kl*Pdc5 and *Km*Pdc5 that had 76% similarity to one another showed only 34% similarity to *Sc*Pdc5. Despite the phylogenetic distance of the yeasts *S. cerevisiae* and *Y. lipolytica*, *Yl*Pdc1 was more similar to the Pdc1 cluster comprising the Pdc1 orthologs from *S. cerevisiae* and *Kluyveromyces* species as well as the *S. cerevisiae* Pdc1 paralogs Pdc5 and Pdc6 than to the other selected proteins.

### 3.2 *In vivo* pyruvate-decarboxylase activity of heterologous enzymes expressed in *S. cerevisiae*

**Fig. 3:**
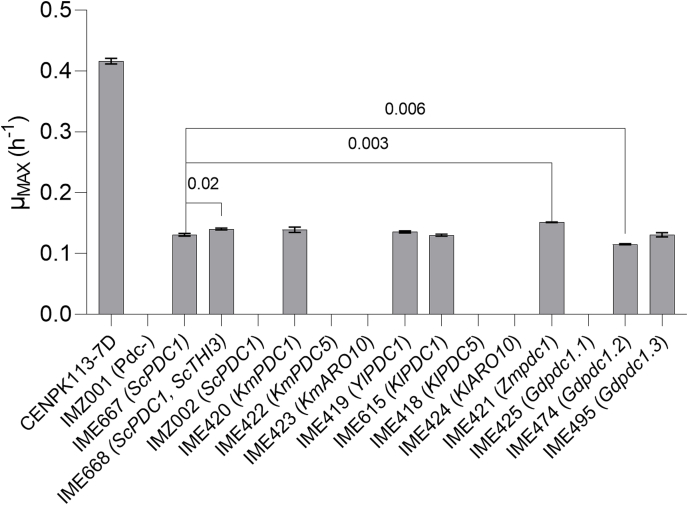
**Specific growth rates of CEN.PK711–7C (*pdc1Δ pdc5Δ pdc6Δ aro10Δ* thi3Δ) expressing individual 2-oxo acid decarboxylases.** Strains IMZ001 (*pdc1Δ pdc5Δ pdc6Δ aro10Δ thi3Δ* 2 *μ*m-*URA3)* and CEN.PK113-7D (*PDC1 PDC5 PDC6 ARO10 THI3*) were used as references. Strains IMZ001, CEN.PK113-7D, IME667 (*ScPDC1↑*), IME668 (*ScPDC1↑ ScTHI3↑*), IMZ002 (*ScARO10↑*), IME615 (*KlPDC1↑*), IME422 (*KmPDC5↑*), IME424 (*KlARO10↑*), IME420 (*KmPDC1↑*), IME423 (*KmARO10↑*), IME418 (*KlPDC5↑*), IME419 (*YlPDC1↑*), IME421 (*Zmpdc1↑*), IME425 (*Gdpdc1.1↑*), IME474 (*Gdpdc1.2↑*) and IME495 (*Gdpdc1.3↑*) expressed different decarboxylases genes from episomal multicopy plasmids and under control of *ScTDH3*pr. For each strain, duplicate cultures were grown on synthetic medium containing 2% glucose (SMD) at 30 °C. Strains with a significantly different (*p* < 0.05, *t*-test) specific growth rate than strain IME667 (*ScPDC1↑*) are indicated with the corresponding *p*-value.

To assess *in vivo* pyruvate decarboxylase activity of the 11 selected enzymes upon introduction in *S. cerevisiae*, they were expressed from an episomal plasmid and under control of the strong consecutive *TDH3* promoter in the pyruvate-decarboxylase-negative strain CENPK711-7C (*ura3Δ pdc1Δ pdc5Δ pdc6Δ aro10Δ thi3Δ*). When precultures on SMEG were transferred to SMD, the empty-vector control strain IMZ001 (CENPK711-7C empty plasmid, *URA3*) and the *Sc*Aro10-expressing strain IMZ002 (CENPK711-7C *ScARO10↑*) failed to grow. This observation was consistent with the inability of pyruvate-decarboxylase-negative *S. cerevisiae* strains to grow on glucose as sole carbon source (Flikweert et al., 1996, 1999). Also strains IME418 (CENPK711-7C *KlPDC5↑*), IME423 (CENPK711-7C *KmARO10↑*), IME424 (CENPK711-7C *KlARO10↑*) and IME425 (CENPK711-7C *Gdpdc1.1↑*), did not show growth on SMD after seven days of incubation, while the same strains were fully grown on SMEG. These results indicated that the heterologous genes introduced into these strains were either not functionally expressed or did not encode a functional pyruvate decarboxylase. In contrast, strains IME419 (CENPK711-7C *YlPDC1↑*), IME615 (CENPK711-7C *KlPDC1↑*), IME420 (CENPK711-7C *KmPDC1↑*), IME421 (CENPK711 *Zmpdc1↑*), IME422 (CENPK711-7C *KmPDC5↑*), IME474 (CENPK711-7C *Gdpdc1.2↑*)*,* IME495 (CENPK711-7C *Gdpdc1.3↑*), and the positive control strain IME667 (CENPK711-7C *ScPDC1↑*) all showed growth without lag-phase on SMD. Specific growth rates on SMD of these strains, including the positive-control strain IME667, were between 0.12 and 0.15 h^−1^. These growth rates were ca. 3-fold lower than that of the reference strain CEN.PK113-7D (0.42 ± 0.00 h^−1^), which retains all 2-oxo acid decarboxylase genes in their native genetic context (Fig. 3).

The platform strain CENPK711-7C used to individually express the 2-oxo acid decarboxylases carried a deletion of *THI3*, a gene that was originally assumed to encode a fifth *S. cerevisiae* 2-oxo acid decarboxylase (Dickinson et al., 1998, 2000; Vuralhan et al., 2003) but was later shown to instead encode a protein involved in thiamine homeostasis (Mojzita and Hohmann, 2006; Nosaka et al., 2008). To investigate if inactivation of *THI3* was responsible for the unexpectedly low specific growth rate of the tested strains, strain IME668 (CEN.PK711–7C *ScPDC1↑ ScTHI3↑*) was constructed. Its specific growth rate on SMD was only 10% higher than that of strain IME667 (CENPK711-7C *ScPDC1↑*) and therefore still much lower than that of strain CEN.PK113-7D (Fig. 3).

### 3.3 *In vitro* comparison of substrate specificity of 2-oxo acid decarboxylase from various origin

**Fig. 4:**
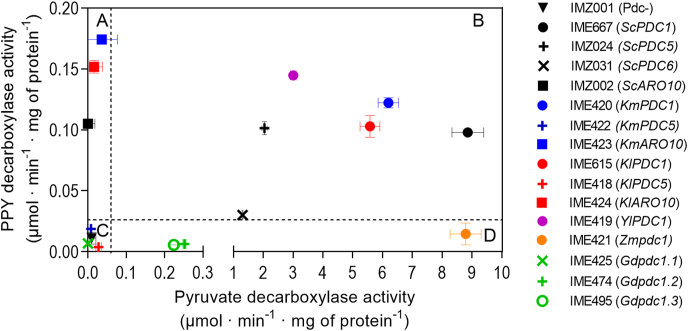
**Specific decarboxylase activities for pyruvate and phenylpyruvate (PPY) in cell extracts of CENPK711-7C (*pdc1Δ pdc5Δ pdc6Δ aro10Δ thi3Δ)* expressing individual 2-oxo acid decarboxylase genes from a multicopy plasmid.** All strains were grown duplicates at 30 °C on synthetic medium containing 2% (v/v) glycerol and 2% (v/v) ethanol as carbon source (SMEG). Black: *S. cerevisiae*, blue: *K. marxianus*, purple; *Y. lipolytica*, red: *K. lactis*, orange; *Z. mobilis,* green: *G. diazotrophicus.* Strains tested were IMZ001 (*pdc1Δ pdc5Δ pdc6Δ aro10Δ thi3Δ URA3↑*), IMZ002 (*ScARO10↑*), IMZ024 (*ScPDC5↑*), IMZ031 (*ScPDC6↑*), IME418 (*KlPDC5↑*), IME419 (*YlPDC1↑*), IME420 (*KmPDC1↑*), IME421 (*Zmpdc1↑*), IME422 (*KmPDC5↑*), IME423 (*KmARO10↑*), IME424 (*KlARO10↑*), IME425 (*Gdpdc1.1↑*), IME474 (*Gdpdc1.2↑*)*,* IME495 (*Gdpdc1.3↑*), IME615 (*KlPDC1↑*) and IME667 (*ScPDC1↑*). The dotted line indicates the detection limit for decarboxylase activity, which was <0.04 μmol mg protein^−1^·min^−1^ for pyruvate as substrate and <20 nmol mg of protein^−1^ min^−1^ for phenylpyruvate as substrate. This results in the visualization of 4 classes: enzymes with decarboxylase activity for A) PPY but not pyruvate, B) both PPY and pyruvate C) no activity for either substrates and D) activity for pyruvate, but not PPY.

To assess the substrate specificities of the selected heterologous pyruvate decarboxylases, enzyme activity assays were performed in cell extracts. In view of the goal of this study to eliminate production of aromatic fusel alcohols and acids, these assays focused on their activities with pyruvate and phenylpyruvate as substrates. Absence of pyruvate decarboxylase activity in cell extracts of strains IMZ002 (*ScARO10↑*), IME423 (*KmARO10↑*), IME424 (*KlARO10↑*), IME418 (*KlPDC5↑*) and IME422 (*KmPDC5↑*) correlated with their inability to grow on SMD. In contrast, cell extracts of strains expressing *Yl*Pdc1 (IME419), *Km*Pdc1 (IME420), *Kl*Pdc1 (IME615), *Zm*Pdc1 (IME421), *Gd*Pdc1.2 (IME474) or *Gd*Pdc1.3 (IME495), as well as the strains expressing *Sc*Pdc1 (IME667), *Sc*Pdc5 (IMZ024) and *Sc*Pdc6 (IMZ031) all showed pyruvate-decarboxylase activities (Fig. 4 and Table S2). The highest activities, above 3 μmol (mg protein)^−1^ min^−1^, were observed in cell extracts of strains expressing yeast Pdc1 orthologs (Fig. 4 and Table S2). Cell extracts of strains expressing either of the two *G. diazotrophicus* decarboxylases (*Gd*Pdc1.2 and *Gd*Pdc1.3) exhibited a 35-fold lower pyruvate-decarboxylase activity than those of a strain expressing *Sc*Pdc1. However, pyruvate-decarboxylase activities of cell extracts of the strain expressing *Zm*Pdc1, the other bacterial pyruvate decarboxylase, were close to those observed with the *Sc*Pdc1-expressing strain IME667 (Fig. 4).

As anticipated, cell extracts of strains expressing yeast Aro10 orthologs showed phenylpyruvate-decarboxylase activity, although activities were two orders of magnitude lower than pyruvate-decarboxylase activities observed in cell extracts of strains expressing yeast or *Z. mobilis* Pdc1 homologs (Fig. 4 and Table S2). Three of the heterologous 2-oxo acid decarboxylases with demonstrated *in vivo* and *in vitro* pyruvate-decarboxylase activity upon expression in *S. cerevisiae* (*Gd*Pdc1.2, *Gd*Pdc1.3 and *Zm*Pdc1) showed no activity with 5 mM phenylpyruvate as substrate (Table 4). These enzymes were therefore identified as promising candidates for replacing the native 2-OADCs in *S. cerevisiae* strains engineered for production of phenylpropanoid (Fig. 4).

**Table 4:** **Specific pyruvate decarboxylase activity, *K***_***m***_**and the Hill coefficient for cell free extracts of *S. cerevisiae* strain CEN.PK711–7C (*pdc1Δ pdc5Δ pdc6Δ aro10Δ thi3Δ)* expressing individual 2-OADC genes.** All strains were grown aerobically at 30 °C, 200 RPM in shake flasks containing 100 mL synthetic medium with 2% w/v ethanol as carbon source (SME). The cell extracts were prepared from late-exponential-phase shake-flask cultures. Different pyruvate concentrations were used as substrate for measuring pyruvate decarboxylase activity ranging from 0.1 mM to 50 mM. Enzyme activities were assayed from duplicate cultures.

Strain	Genotype	*K*_*m*_ (mM) ± SD	V_MAX_ (μmol min^−1^ (mg of protein)^−1^ ± SD	Hill coefficient ± SD	V_MAX_/*K*_*m*_ ratio
IME667	*ScPDC1*	2.5 ± 0.0	11.2 ± 0.0	2.4 ± 0.1	4.5
IME420	*KmPDC1*	2.9 ± 0.1	7.8 ± 0.1	2.4 ± 0.1	2.7
IME419	*Ylpdc1*	1.3 ± 0.0	2.5 ± 0.0	1.3 ± 0.0	1.9
IME615	*Klpdc1*	3.1 ± 0.1	9.9 ± 0.1	2.3 ± 0.0	3.2
IME421	*Zmpdc1*	0.6 ± 0.0	5.4 ± 0.1	1.3 ± 0.1	8.8
IME474	*Gdpdc1.2*	0.8 ± 0.1	0.4 ± 0.0	1.0 ± 0.0	0.5
IME495	*Gdpdc1.3*	0.8 ± 0.0	0.2 ± 0.0	1.0 ± 0.0	0.3

*V*max and *Km* values were obtained by performing a nonlinear regression of specific decarboxylase activity over the substrate concentration using either a Michaelis-Menten model or a sigmoidal allosteric model. The Hill coefficients were calculated using the Hill equation; A Hill coefficient of 2.0 indicates positive cooperativity.

To estimate the Michaelis constant (*K*_*m*_) of the heterologous pyruvate decarboxylases for pyruvate, enzyme activity assays with cell extracts of strains expressing the prokaryotic enzymes and *Sc*Pdc1 yeast orthologs (*Kl*Pdc1, *Km*Pdc1 and *Yl*Pdc1) were performed at pyruvate concentrations ranging from 0.1 to 50 mM (Fig. S1). To investigate whether, similar to *S. cerevisiae* pyruvate decarboxylase (Hübner et al., 1978), the heterologous pyruvate decarboxylases exhibit cooperativity, the data was fitted by non-linear regression to substrate-saturation Michaelis-Menten kinetics as well as to sigmoidal allosteric Hill kinetics (Table 4). Consistent with literature (Romagnoli et al., 2012), cell extracts containing *Sc*Pdc1 showed a Hill coefficient of 2.4, while a similar cooperativity was observed for cell extracts containing the *Kluyveromyces* enzymes *Kl*Pdc1 and *Km*Pdc1. In contrast, assays with cell extracts containing either *Y. lipolytica* Pdc1 or one of the three bacterial enzymes (*Zm*pdc1, *Gd*pdc1.2 or *Gd*pdc1.3), yielded a Hill coefficient close to one and absence of a sigmoidal relation between substrate concentration and enzyme activity (Table 4, Fig. S1), thus indicating absence of cooperativity (Table 4). In these assays, the *Z. mobilis* pyruvate decarboxylase *Zm*Pdc1 showed a 4-fold lower *K*_*m*_ than *Sc*Pdc1 and a higher V_max_/*K*_*m*_ ratio than the *G. diazotrophicus* pyruvate decarboxylases.

### 3.4 Decarboxylase swapping in a coumaric acid-producing *S. cerevisiae* strain

To investigate whether replacement of the native yeast 2-OADCs (Pdc1, Pdc5, Pdc6 and Aro10) by heterologous pyruvate decarboxylases (‘decarboxylase swapping’) can eliminate formation of by-products in *S. cerevisiae* strains engineered for phenylpropanoid production, a tester strain producing coumaric acid was constructed. To this end, *ARO10, PDC5* and *PDC6* were first deleted from the previously constructed strain IMX1593, which overexpresses feedback-insensitive alleles of the DAHP synthase and chorismate mutase (*aro3Δ ARO4*^*K229L*^↑ *ARO7*^*T226I*^↑) (Hassing et al., 2019). Subsequently, expression cassettes for *PlstlA, Rctal1t*, *AtC4H and AtCPR1*, which encode for respectively, a phenylalanine ammonia lyase, tyrosine ammonia lyase, cinnamic acid hydroxylase and its cytochrome p450 reductase, required for the activation of the cytochrome P450, were integrated at the X3 locus on CHRX (Mikkelsen et al., 2012). Deletion of the pyruvate decarboxylase gene *PDC1* yielded the 2-OADC-negative, coumaric acid producing platform strain IMX2668. This strain was transformed with multi-copy plasmids carrying expression cassettes for the different 2-oxo acid decarboxylases with specificity for pyruvate. All these strains grew on SMD in shake-flask cultures, albeit slower than the *ScPDC1-*expressing reference strain (IME677) (Table 5). Consistent with their low pyruvate-decarboxylase activities in cell extracts, the lowest specific growth rates were observed for the two strains expressing the *G. diazotrophicus* pyruvate decarboxylases (Table 5).

**Table 5:** **Specific growth rates of IMX2668, a coumaric acid producing background strain, fully devoid of all native 2-OADCS (*pdc1Δ, pdc5Δ, pdc6Δ, aro10Δ*), expressing individual 2-OADC genes from a multicopy plasmid.** All strains were grown aerobically in biological duplicates at 30 °C, 200 RPM in shake flasks containing 100 mL synthetic medium with 2% w/v glucose as carbon source (SMD). CEN.PK113-7D (*PDC1 PDC5 PDC6 ARO10*), IME656 (pGGKd017 (*URA3↑*), IME677 (*ScPDC1↑*), IME658 (*Zmpdc1↑*), IME659 (*KmPDC1↑*), IME660 (*YlPDC1↑*), IME661 (*KlPDC1↑*), IME662 (*Gdpdc1.2↑*) and IME663 (*Gdpdc1.3↑*).

Strain	Genotype	μ_MAX_ (h^−1^)
CEN.PK113-7D	Ref.	0.39 ± 0.00
IME656	IMX2668 *URA3↑*	0.00 ± 0.00
IME677	IMX2668 *ScPDC1↑*	0.27 ± 0.00
IME658	IMX2668 *Zmpdc1↑*	0.20 ± 0.00
IME659	IMX2668 *KmPDC1↑*	0.28 ± 0.00
IME660	IMX2668 *YlPDC1↑*	0.18 ± 0.00
IME661	IMX2668 *KlPDC1↑*	0.23 ± 0.00
IME662	IMX2668 *Gdpdc1.2↑*	0.11 ± 0.00
IME663	IMX2668 *Gdpdc1.3↑*	0.17 ± 0.00

**Fig. 5:**
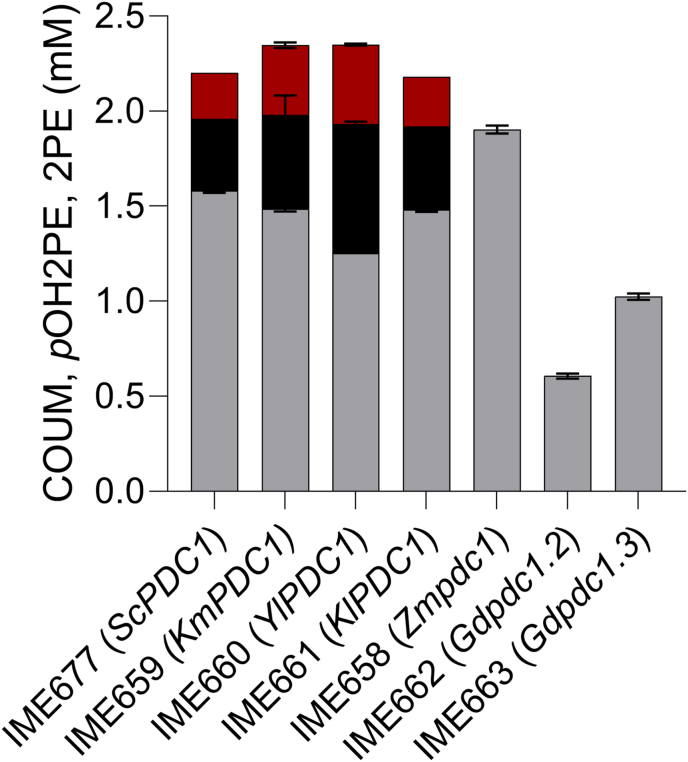
**Metabolite profile of the coumaric acid producing reference strain IMX2668 (*Scpdc1Δ, Scpdc5Δ, Scpdc6Δ, Scaro101Δ*) expressing individual 2-OADC genes from a multicopy vector.** IME677 (*ScPDC1↑*), IME658 (*Zmpdc1↑*) IME659 (*KmPDC1↑*), IME660 (*YlPDC1↑*), IME661 (*KlPDC1↑*), IME662 (*Gdpdc1.2↑*) and IME663 (*Gdpdc1.3↑*) were grown at 30 °C in biological triplicates on synthetic medium containing glucose as sole carbon source (SMD). All strains were inoculated at OD_660_ = 0.2 and grown for 72 h until they reached stationary phase. Red: 2-phenylethanol (2 PE), black: *p*-hydroxyphenylethanol (*p*OH2PE), grey: coumaric acid (COUM). The concentrations of ethanol, glucose, pyruvate and glycerol are depicted in Fig. S2.

In line with the results of the enzyme activity assays, the coumaric acid-producing strains IME659 (*KmPDC1↑*), IME660 (*YlPDC1↑*) and IME661 (*KlPDC1↑*) produced 2-phenylethanol and *p*-hydroxyphenylethanol, at concentrations ranging from 0.26 mM to 0.69 mM (Fig. 5). In contrast, strains IME658 (*Zmpdc1↑*), IME662 (*Gdpdc1.2↑*) and IME663 (*Gdpdc1.3↑*) did not show detectable concentrations of these aromatic fusel alcohols. Cultures of IME658 (*Zmpdc1↑*) reached 20% higher final coumaric acid concentration than the reference strain IME677 (*ScPDC1↑*). In the shake-flask cultures, strains IME662 (*Gdpdc1.2↑*) and IME663 (*Gdpdc1.3↑*), did not consume all glucose and did not produce detectable amounts of ethanol (Fig. S2).

**Fig. 6:**
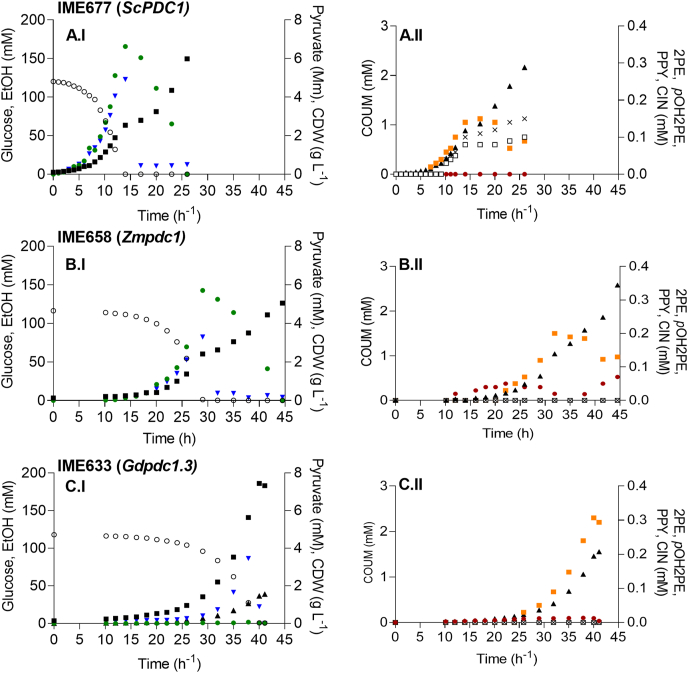
**Aerobic batch cultivation in bioreactors of coumaric acid producing strains, expressing *ScPDC1, Kmpdc1* or *Gdpdc1.3* as sole pyruvate decarboxylase from a multicopy vector.** All strains were grown aerobically at 30 °C, pH 5.0, in biological duplicates on synthetic medium containing glucose as sole carbon source (SMD). The results shown are from a single representative cultivation. Panels A: control strain IME677 (*ScPDC1↑*), panels B: IME658 (*Zmpdc1↑*) and panels C: IME663 (*Gdpdc1.3↑*). The left column (I) depicts the concentration of the cell dry weight (CDW) [■ (g L^−1^)], glucose [○ (mM)], ethanol [● (mM); EtOH] and pyruvate [▾ (mM); PYR] over time whereas the right column (II) shows the concentration of the aromatic metabolites coumaric acid [▴ (mM); COUM], cinnamic acid [● (mM); CIN], 2-phenylethanol [✕ (mM); 2 PE], *p*-hydroxyphenylethanol [□ (mM); pOH2PE] and phenylpyruvate [■ (mM); PPY].

To more accurately quantify the impact of decarboxylase swapping on coumaric acid production, the coumaric acid-producing reference strain IME677 (*ScPDC1↑*), as well as strains IME658 (*Zmpdc1↑*) and IME663 (*Gdpdc1.3↑*) were grown aerobically on SMD in pH-controlled bioreactors. Under these conditions, the reference strain IME677 produced 2.2 mM of coumaric acid and displayed the typical diauxic growth pattern of aerobic glucose-grown batch cultures of *S. cerevisiae*, with an initial respiro-fermentative growth phase followed by a respiratory ethanol consumption phase (De Deken, 1966) (Fig. 6A.I). As observed in shake-flask cultures, strain IME677 (*ScPDC1↑*) produced 2-phenylethanol (0.10 mM) and *p*-hydroxyphenylethanol (0.15 mM) (Fig. 6A.II), which together corresponded to 12% of the total extracellular aromatic metabolites. In addition, this strain excreted detectable amounts of phenylpyruvate (0.10 mM) (Fig. 6A.II).

Although growing 30% slower than strain IME677, strain IME658 (*Zmpdc1↑*) also displayed a respiro-fermentative growth (Fig. 6B.I). However, in contrast to the *Sc*Pdc1-expressing reference strain, this *Zmpdc1*-expressing strain did not produce detectable levels of aromatic fusel alcohols (Fig. 6B.II). As in strain IME677, phenylpyruvate was detected (0.12 mM). In comparison with strain IME677, strain IME658 showed a higher coumaric acid titer (2.5 mM vs 2.2 mM) and, in contrast to the reference strain, secreted the coumaric acid precursor cinnamic acid (0.1 mM). In the absence of aromatic fusel alcohol production, strain IME658 (*Zmpdc1↑*) therefore excreted 14% more coumaric acid and 24% more coumaric acid precursors than the reference strain IME677 (*ScPDC1↑*). These improvements were also observed in the molar yields of coumaric acid on glucose (Y_COUM/S_) in these strains, which were 22.0 ± 0.2 mmol mol^−1^ for strain IME658 and 18.4 ± 0.7 mmol mol^−1^ for strain IME677 (Table 6).

**Table 6:** **Performance of aerobic batch cultures of 2-OADC expressing, coumaric acid producing strains.** Specific growth rate (μ) and yields (Y) of biomass (X) and ethanol (EtOH) on glucose (S) during the glucose phase (a), the yields of biomass (X), 2-phenylethanol (2 PE), *p*-hydroxyphenylethanol (*p*OH2PE) and coumaric acid (COUM) on glucose during the entire cultivation and the accumulated titer of all measured aromatics (2 PE, *p*OH2PE, COUM, phenylpyruvate (PPY) and cinnamic acid (CIN)) of the *S. cerevisiae* strains IME677 (control strain, *ScPDC1↑*), IME658 (*Zmpdc1↑*) and IME633 (*Gdpdc1.3↑*).

Strain	IME677	IME658	IME663
Relevant genotype	*ScPDC1*	*Zmpdc1*	*Gdpdc1.3*
aμ (h^−1^)	0.29 ± 0.01	0.20 ± 0.02	0.14 ± 0.00
aY_X/S_ (g g^−1^)	0.12 ± 0.00	0.11 ± 0.00	0.35 ± 0.00
aY_EtOH/S_ (mol mol^−1^)	1.37 ± 0.01	1.20 ± 0.05	0.00 ± 0.00
			
Y_X/S_ (g g^−1^)	0.27 ± 0.01	0.24 ± 0.01	0.35 ± 0.00
Y_2PE/S_ (mmol mol^−1^)	1.08 ± 0.16	0.00 ± 0.00	0.00 ± 0.00
Y_*p*OH2PE/S_ (mmol mol^−1^)	1.29 ± 0.04	0.00 ± 0.00	0.00 ± 0.00
Y_COUM/S_ (mmol mol^−1^)	18.39 ± 0.50	21.97 ± 0.26	12.90 ± 0.48
			
∑Aromatics (mM)	2.58 ± 0.11	2.66 ± 0.08	1.78 ± 0.10

a Determined from the glucose phase only.

Strain IME663 (*Gdpdc1.3↑*) showed a 50% lower specific growth rate in the bioreactor cultures than strain IME677 (*ScPDC1↑*) (Table 6). In comparison to the other two coumaric acid-producing strains, it did not produce detectable amounts of ethanol and reached 30% higher final biomass concentrations. In contrast to the shake-flask cultures of strain IME663, the bioreactor cultures consumed all glucose. Although aromatic fusel alcohols were not detected in culture supernatants, strain IME663 reached a lower coumaric acid titer than strain IME677 (1.5 mM vs 2.2 mM) and, additionally, produced nearly three-fold higher extracellular phenylpyruvate concentrations (0.3 mM).

## 4 Discussion

Microbial thiamine-pyrophosphate-dependent pyruvate decarboxylases (EC 4.1.1.1) exhibit different kinetic properties and substrate specificities (Vuralhan et al., 2005; Romagnoli et al., 2012; Milne et al., 2015; Stribny et al., 2016). By exploring this diversity, we identified bacterial pyruvate decarboxylases that did not, or very slowly, decarboxylate phenylpyruvate *in vitro* and could functionally replace the native *S. cerevisiae* pyruvate decarboxylases in glucose-grown cultures *in vivo*. Replacing all native *S. cerevisiae* 2-OADCs in a coumaric acid producing strain by bacterial decarboxylases from *G. diazotrophicus* or *Z. mobilis* eliminated formation of aromatic by-products via the Ehrlich pathway. Moreover, the coumaric acid-producing strain *S. cerevisiae* IME658, in which the native yeast 2-OADCs were replaced by *Z. mobilis pdc1,* did not produce aromatic fusel alcohols and showed a higher coumaric acid yield than the congenic strain IME677 that instead expressed *ScPDC1*.

While our study provided a clear proof of principle for the ‘decarboxylase swapping’ approach, the *Zmpdc1*-expressing strain grew 30% slower than the *ScPDC1* expressing strain. These different growth rates occurred despite high and similar pyruvate-decarboxylase activities in cell extracts of SMEG-grown cultures of *pdc1Δ pdc5Δ pdc6Δ aro10Δ thi3Δ* strains carrying the same *Zmpdc1* and *ScPDC1* expression vectors (Table S2 and Fig. 4). Slower growth of the *Zmpdc1*-expressing strain may be related to a reported 20-fold higher sensitivity of *Zm*Pdc1 to inhibition by its product acetaldehyde (Goetz et al., 2001). It would therefore be interesting to express the acetaldehyde-tolerant variant *Zm*Pdc1^W392M^ (Bruhn et al., 1995; Yun and Kim, 2008). Alternatively, as proposed earlier for a *ScPDC1*-overexpressing *S. cerevisiae* strain (van Hoek et al., 1998), reduced growth rates on SMD of strains expressing pyruvate-decarboxylase genes from episomal-multicopy plasmids may reflect protein-burden effects. Further metabolic engineering and/or adaptive laboratory evolution (Mans et al., 2018) can be applied to identify optimal expression levels of these pyruvate-decarboxylases. When impacts on specific growth rate can be prevented, the 2-oxo acid decarboxylase swapping strategy outlined in this study should be applicable for reduction of by-product formation by yeast strains engineered for production of a wide range phenylpropanoids including stilbenoids, flavonoids and hydroxycinnamic acids.

Applicability of *Zm*Pdc1 in yeast biotechnology may extend beyond prevention of aromatic by-product formation. Previous research showed that, in contrast to *Sc*Pdc1 (Romagnoli et al., 2012), *Z. mobilis* pyruvate decarboxylase does not decarboxylate the 2-oxo acids 3-methyl-2-oxopentanoate, 4-methyl-2-oxopentanoate and 3-methyl-2-oxobutanoate (Chang et al., 2000; Siegert et al., 2005), which are derived from isoleucine, leucine and valine, respectively. Elimination of these volatile fusel alcohol by-products during yeast-based ethanol production may enable reduced processing costs (Mayer et al., 2015).

Expression of 2-OADCs from an episomal-multicopy plasmid in the CEN.PK711–7C background (*pdc1*Δ *pdc5*Δ *pdc6*Δ *aro10*Δ *thi3*Δ) resulted in specific growth rates for all strains between 0.12 and 0.15 h^−1^ (Fig. 3). When CEN.PK113-7D is grown on synthetic medium with glucose as carbon source and ammonium sulphate as nitrogen source, *ScPDC1* transcript levels are 10 fold higher compared to the other decarboxylases (Vuralhan et al., 2005) and serves as the main decarboxylase under these conditions. However, IME667 (*ScPDC1↑*) only had a specific growth rate of 0.13 h^−1^ whereas a specific growth rate close to CEN.PK113-7D (0.42 h^−1^) was expected. Simultaneous expression of *ScPDC1* and *ScTHI3* (IME668), did not lead to a higher growth rate. Surprisingly, expression of the same 2-OADC multicopy plasmids in a newly constructed, *pdc1*Δ *pdc5*Δ *pdc6*Δ *aro10*Δ coumaric acid producing strain (IMX2668) resulted in strains with much higher specific growth rates (0.11–0.28 h^−1^ (Table 5)). Therefore a genetic defect, besides the *THI3* deletion, in the CEN.PK711–7C background and its transformants is causing the low specific growth which may find its origin in the use loxP-Cre recombinase during the construction of the strain (Romagnoli et al., 2012); a method that can cause chromosomal recombination (Solis-Escalante et al., 2015). Whole genome sequencing might elucidate the exact cause.

In addition to outlining a metabolic engineering strategy for minimizing by-product formation, our results provided new insights in the diversity of microbial 2-OADCs. Except for *Sc*Aro10, *Km*Aro10 and *Kl*Aro10, the genes evaluated in this study were annotated as structural genes encoding pyruvate decarboxylases. Based on the inability of *Km*Pdc5 and *Km*Pdc5 to complement the growth defect of a pyruvate-decarboxylase-negative *S. cerevisiae* strain and the absence of *in vitro* decarboxylase activity with pyruvate or phenylpyruvate, further research is required to investigate their catalytic activity. A predicted pyruvate decarboxylase (*Yl*Pdc1*;* YALI0D10131g) in *Y. lipolytica*, which showed low sequence similarity with other yeast pyruvate decarboxylases, is active and in contrast to other yeast pyruvate-decarboxylases (König et al., 2009) did not exhibit cooperativity for its substrate (Table 5). This result is intriguing in view of the inability of this yeast to produce ethanol (Gatter et al., 2016) and because it is generally assumed that, in *Y. lipolytica*, cytosolic acetyl-CoA, which is a key precursor for lipid synthesis by this oleaginous yeast, originates from the activity of the ATP-citrate lyase (Zhang et al., 2016). Combined with previously reported aldehyde dehydrogenases (Dujon et al., 2004) and an acetyl-CoA synthetase (Kujau et al., 1992; Gatter et al., 2016), *Yl*Pdc1 could provide an alternative, energetically less efficient (van Rossum et al., 2016), bypass. Further research should establish the physiological relevance of *Yl*Pdc1 in its native host.

The present study, which explored only a fraction of the natural diversity of 2-OADCs, illustrates for further screening, mutagenesis and targeted protein engineering to tailor catalytic and regulatory properties of these key enzymes to specific applications in biotechnology.

## Financial support

This project has received funding from the European Union’s Horizon 2020 Research and Innovation program under grant agreement No 720824.

## Declaration of competing interest

None.

## Appendix A Supplementary data

Multimedia component 4 Multimedia component 4

figs1 figs1

figs2 figs2
