# Supplementary material for: Elimination of aromatic fusel alcohols as by-products of Saccharomyces cerevisiae strains engineered for phenylpropanoid production by 2-oxo-acid decarboxylase replacement
Source: Metab Eng Commun. 2021 Sep 7;13:e00183. doi: 10.1016/j.mec.2021.e00183 (PMC8450241; doi:10.1016/j.mec.2021.e00183)
Supplement: Multimedia component 1 [file mmc1.docx]

**Data set S1. The amino acid sequences of all 2-oxo acid decarboxylases used in this study or references.**

**>*ScPDC1* (CEN.PK113-7D)**

MSEITLGKYLFERLKQVNVNTVFGLPGDFNLSLLDKIYEVEGMRWAGNAN

ELNAAYAADGYARIKGMSCIITTFGVGELSALNGIAGSYAEHVGVLHVVG

VPSISSQAKQLLLHHTLGNGDFTVFHRMSANISETTAMITDIATAPAEID

RCIRTTYVTQRPVYLGLPANLVDLNVPAKLLQTPIDMSLKPNDAESEKEV

IDTILALVKDAKNPVILADACCSRHDVKAETKKLIDLTQFPAFVTPMGKG

SIDEQHPRYGGVYVGTLSKPEVKEAVESADLILSVGALLSDFNTGSFSYS

YKTKNIVEFHSDHMKIRNATFPGVQMKFVLQKLLTNIADAAKGYKPVAVP

ARTPANAAVPASTPLKQEWMWNQLGNFLQEGDVVIAETGTSAFGINQTTF

PNNTYGISQVLWGSIGFTTGATLGAAFAAEEIDPKKRVILFIGDGSLQLT

VQEISTMIRWGLKPYLFVLNNDGYTIEKLIHGPKAQYNEIQGWDHLSLLP

TFGAKDYETHRVATTGEWDKLTQDKSFNDNSKIRMIEVMLPVFDAPQNLV

EQAKLTAATNAKQ

**>*ScPDC6* (CEN.PK113-7D)**

MSEITLGKYLFERLKQVNVNTIFGLPGDFNLSLLDKIYEVDGLRWAGNAN

ELNAAYAADGYARIKGLSVLVTTFGVGELSALNGIAGSYAEHVGVLHVVG

VPSISAQAKQLLLHHTLGNGDFTVFHRMSANISETTSMITDIATAPSEID

RLIRTTFITQRPSYLGLPANLVDLKVPGSLLEKPIDLSLKPNDPEAEKEV

IDTVLELIQNSKNPVILSDACASRHNVKKETQKLIDLTQFPAFVTPLGKG

SIDEQHPRYGGVYVGTLSKPDVKQAVESADLILSVGALLSDFNTGSFSYS

YKTKNVVEFHSDYVKVKNATFPGVQMKFALQNLLKVIPDVVKGYKSVPVP

TKTPANKGVPASTPLKQEWLWNELSKFLQEGDVIISETGTSAFGINQTIF

PKDAYGISQVLWGSIGFTTGATLGAAFAAEEIDPNKRVILFIGDGSLQLT

VQEISTMIRWGLKPYLFVLNNDGYTIEKLIHGPHAEYNEIQTWDHLALLP

AFGAKKYENHKIATTGEWDALTTDSEFQKNSVIRLIELKLPVFDAPESLI

KQAQLTAATNAKQ

**>*ScPDC5* (CEN.PK113-7D)**

MSEITLGKYLFERLSQVNCNTVFGLPGDFNLSLLDKLYEVKGMRWAGNAN

ELNAAYAADGYARIKGMSCIITTFGVGELSALNGIAGSYAEHVGVLHVVG

VPSISSQAKQLLLHHTLGNGDFTVFHRMSANISETTAMITDIANAPAEID

RCIRTTYTTQRPVYLGLPANLVDLNVPAKLLETPIDLSLKPNDAEAEAEV

VRTVVELIKDAKNPVILADACASRHDVKAETKKLMDLTQFPVYVTPMGKG

AIDEQHPRYGGVYVGTLSRPEVKKAVESADLILSIGALLSDFNTGSFSYS

YKTKNIVEFHSDHIKIRNATFPGVQMKFALQKLLDAIPEVVKDYKPVAVP

ARVPITKSTPANTPMKQEWMWNHLGNFLREGDIVIAETGTSAFGINQTTF

PTDVYAIVQVLWGSIGFTVGALLGATMAAEELDPKKRVILFIGDGSLQLT

VQEISTMIRWGLKPYIFVLNNNGYTIEKLIHGPHAEYNEIQGWDHLALLP

TFGARNYETHRVATTGEWEKLTQDKDFQDNSKIRMIEVMLPVFDAPQNLV

KQAQLTAATNAKQ

**>*ScPDC*1 S288C**

MSEITLGKYLFERLKQVNVNTVFGLPGDFNLSLLDKIYEVEGMRWAGNAN

ELNAAYAADGYARIKGMSCIITTFGVGELSALNGIAGSYAEHVGVLHVVG

VPSISAQAKQLLLHHTLGNGDFTVFHRMSANISETTAMITDIATAPAEID

RCIRTTYVTQRPVYLGLPANLVDLNVPAKLLQTPIDMSLKPNDAESEKEV

IDTILALVKDAKNPVILADACCSRHDVKAETKKLIDLTQFPAFVTPMGKG

SIDEQHPRYGGVYVGTLSKPEVKEAVESADLILSVGALLSDFNTGSFSYS

YKTKNIVEFHSDHMKIRNATFPGVQMKFVLQKLLTTIADAAKGYKPVAVP

ARTPANAAVPASTPLKQEWMWNQLGNFLQEGDVVIAETGTSAFGINQTTF

PNNTYGISQVLWGSIGFTTGATLGAAFAAEEIDPKKRVILFIGDGSLQLT

VQEISTMIRWGLKPYLFVLNNDGYTIEKLIHGPKAQYNEIQGWDHLSLLP

TFGAKDYETHRVATTGEWDKLTQDKSFNDNSKIRMIEIMLPVFDAPQNLV

EQAKLTAATNAKQ

**>*ScPDC6* S288C**

MSEITLGKYLFERLKQVNVNTIFGLPGDFNLSLLDKIYEVDGLRWAGNAN

ELNAAYAADGYARIKGLSVLVTTFGVGELSALNGIAGSYAEHVGVLHVVG

VPSISAQAKQLLLHHTLGNGDFTVFHRMSANISETTSMITDIATAPSEID

RLIRTTFITQRPSYLGLPANLVDLKVPGSLLEKPIDLSLKPNDPEAEKEV

IDTVLELIQNSKNPVILSDACASRHNVKKETQKLIDLTQFPAFVTPLGKG

SIDEQHPRYGGVYVGTLSKQDVKQAVESADLILSVGALLSDFNTGSFSYS

YKTKNVVEFHSDYVKVKNATFLGVQMKFALQNLLKVIPDVVKGYKSVPVP

TKTPANKGVPASTPLKQEWLWNELSKFLQEGDVIISETGTSAFGINQTIF

PKDAYGISQVLWGSIGFTTGATLGAAFAAEEIDPNKRVILFIGDGSLQLT

VQEISTMIRWGLKPYLFVLNNDGYTIEKLIHGPHAEYNEIQTWDHLALLP

AFGAKKYENHKIATTGEWDALTTDSEFQKNSVIRLIELKLPVFDAPESLI

KQAQLTAATNAKQ

**>*ScPDC5* S288C**

MSEITLGKYLFERLSQVNCNTVFGLPGDFNLSLLDKLYEVKGMRWAGNAN

ELNAAYAADGYARIKGMSCIITTFGVGELSALNGIAGSYAEHVGVLHVVG

VPSISSQAKQLLLHHTLGNGDFTVFHRMSANISETTAMITDIANAPAEID

RCIRTTYTTQRPVYLGLPANLVDLNVPAKLLETPIDLSLKPNDAEAEAEV

VRTVVELIKDAKNPVILADACASRHDVKAETKKLMDLTQFPVYVTPMGKG

AIDEQHPRYGGVYVGTLSRPEVKKAVESADLILSIGALLSDFNTGSFSYS

YKTKNIVEFHSDHIKIRNATFPGVQMKFALQKLLDAIPEVVKDYKPVAVP

ARVPITKSTPANTPMKQEWMWNHLGNFLREGDIVIAETGTSAFGINQTTF

PTDVYAIVQVLWGSIGFTVGALLGATMAAEELDPKKRVILFIGDGSLQLT

VQEISTMIRWGLKPYIFVLNNNGYTIEKLIHGPHAEYNEIQGWDHLALLP

TFGARNYETHRVATTGEWEKLTQDKDFQDNSKIRMIEVMLPVFDAPQNLV

KQAQLTAATNAKQ

**>*KlPDC1* (CBS 2359)**

MSEITLGRYLFERLKQVEVQTIFGLPGDFNLSLLDNIYEVPGMRWAGNAN

ELNAAYAADGYARLKGMSCIITTFGVGELSALNGIAGSYAEHVGVLHVVG

VPSVSSQAKQLLLHHTLGNGDFTVFHRMSSNISETTAMITDINTAPAEID

RCIRTTYVSQRPVYLGLPANLVDLTVPASLLDTPIDLSLKPNDPEAEEEV

IENVLQLIKEAKNPVILADACCSRHDAKAETKKLIDLTQFPAFVTPMGKG

SIDEKHPRFGGVYVGTLSSPAVKEAVESADLVLSVGALLSDFNTGSFSYS

YKTKNIVEFHSDYTKIRSATFPGVQMKFALQKLLTKVADAAKGYKPVPVP

SEPEHNEAVADSTPLKQEWVWTQVGEFLREGDVVITETGTSAFGINQTHF

PNNTYGISQVLWGSIGFTTGATLGAAFAAEEIDPKKRVILFIGDGSLQLT

VQEISTMIRWGLKPYLFVLNNDGYTIERLIHGETAQYNCIQNWQHLELLP

TFGAKDYEAVRVSTTGEWNKLTTDEKFQDNTRIRLIEVMLPTMDAPSNLV

KQAQLTAATNAKN

**>*KlPDC5* (CBS 2359)**

METKTLIHSGAAKEMSYTERYNVAPLIPLPEYLFHRLFQLNCRTVFGVAN

YSTAKLYQAIAASGIQWIQTINQLNTSFAVDAYGRAIGVSCYVTSESAEL

GHVNGFFGSFCEYVPILQVVVLEQSHDLERLIGDVSIFHDVVDDPSEIDS

CVRTLFWGKRPVYMGLRSKDATKLVPSSSLNGNIADKMGIKNTFFQTDTI

KRVIDKIIAEVYASSRPLIVVDALIDRYNYNSTIQNFLTETGIPFVTTLM

SKGSIDESLPNFVGTFLGTMSQPIVREYMNNADCTLILGCMIENFKNSYC

RFNYKSKNQILLWNDRVKIENNIIPDILLHELLPQLIASLDTTKIVNSRP

VTIPNMIPRVEPQPVTFLRQEYLWFKMSTWLKQGDVIISESGTSAIGLLQ

QKFPDNTRLVSQAIWNSSGYSIGACLGILAAYRDMGTLDKHRIILMVGDG

SLQFTFQELSTILTHGFKPYIFVINNQGYTVDRTLNREKTHLNATYFDIQ

PWELLKLPSLFYSQEYFKRRCMSVGELNSLLSDKEFNKSDQLKIVELILP

SMDVPVLLDPRDDSSDDESSPQHKRPRT

**>*YlPDC1***

MSDSEPQMVDLGDYLFARFKQLGVDSVFGVPGDFNLTLLDHVYNVDMRWV

GNTNELNAGYSADGYSRVKRLACLVTTFGVGELSAVAAVAGSYAEHVGVV

HVVGVPSTSAENKHLLLHHTLGNGDFRVFAQMSKLISEYTHHIEDPSEAA

DVIDTAIRIAYTHQRPVYIAVPSNFSEVDIADQARLDTPLDLSLQPNDPE

SQYEVIEEICSRIKAAKKPVILVDACASRYRCVDETKELAKITNFAYFVT

PMGKGSVDEDTDRYGGTYVGSLTAPATAEVVETADLIISVGALLSDFNTG

SFSYSYSTKNVVELHSDHVKIKSATYNNVGMKMLFPPLLEAVKKLVAETP

DFASKALAVPDTTPKIPEVPDDHITTQAWLWQRLSYFLRPTDIVVTETGT

SSFGIIQTKFPHNVRGISQVLWGSIGYSVGAACGASIAAQEIDPQQRVIL

FVGDGSLQLTVTEISCMIRNNVKPYIFVLNNDGYTIERLIHGENASYNDV

HMWKYSKILDTFNAKAHESIVVNTKGEMDALFDNEEFAKPDKIRLIEVMC

DKMDAPASLIKQAELSAKTNV

**>*KmPDC1* (NRBC 1777)**

MSEITLGRYLFERLKQVEVQTIFGLPGDFNLSLLDKIYEVPGMRWAGNAN

ELNAAYAADGYARLKGMACVITTFGVGELSALNGIAGSYAEHVGVLHVVG

VPSISSQAKQLLLHHTLGNGDFTVFHRMSSNISETTAMITDINSAPSEID

RCIRTTYISQRPVYLGLPANLVDLKVPASLLETPIDLSLKPNDPEAENEV

LETVLELIKDAKNPVILADACCSRHNVKAETKKLIDITQFPAFVTPMGKG

SIDEQHPRFGGVYVGTLSSPEVKEAVESADLVLSVGALLSDFNTGSFSYS

YKTKNIVEFHSDYIKVRNATFPGVQMKFVLQKLLTKVKDAAKGYKPVPVP

HAPRDNKPVADSTPLKQEWVWTQVGKFLQEGDVVLTETGTSAFGINQTHF

PNDTYGISQVLWGSIGFTGGATLGAAFAAEEIDPKKRVILFIGDGSLQLT

VQEISTMIRWGLKPYLFVLNNDGYTIERLIHGETAQYNCIQSWKHLDLLP

TFGAKDYEAVRVATTGEWNKLTTDKKFQENSKIRLIEVMLPVMDAPSNLV

KQAQLTASINAKQE

>***Zmpdc1 (Zymomonas mobilis subsp. mobilis* ATCC 10988*)***

MSYTVGTYLAERLVQIGLKHHFAVAGDYNLVLLDNLLLNKNMEQVYCCNE

LNCGFSAEGYARAKGAAAAVVTYSVGALSAFDAIGGAYAENLPVILISGA

PNNNDHAAGHVLHHALGKTDYHYQLEMAKNITAAAEAIYTPEEAPAKIDH

VIKTALREKKPVYLEIACNIASMPCAAPGPASALFNDEASDEASLNAAVE

ETLKFIANRDKVAVLVGSKLRAAGAEEAAVKFADALGGAVATMAAAKSFF

PEENPHYIGTSWGEVSYPGVEKTMKEADAVIALAPVFNDYSTTGWTDIPD

PKKLVLAEPRSVVVNGIRFPSVHLKDYLTRLAQKVSKKTGALDFFKSLNA

GELKKAAPADPSAPLVNAEIARQVEALLTPNTTVIAETGDSWFNAQRMKL

PNGARVEYEMQWGHIGWSVPAAFGYAVGAPERRNILMVGDGSFQLTAQEV

AQMVRLKLPVIIFLINNYGYTIEVMIHDGPYNNIKNWDYAGLMEVFNGNG

GYDSGAGKGLKAKTGGELAEAIKVALANTDGPTLIECFIGREDCTEELVK

WGKRVAAANSRKPVNKLL

**>*Gdpdc1.1* (Sequence van Zyl et al)**

MTYTVGRYLADRLAQIGLKHHFAVAGDYNLVLLDQLLLNTDMQQIYCSNE

LNCGFSAEGYARANGAAAAIVTFSVGALSAFNALGGAYAENLPVILISGA

PNANDHGTGHILHHTLGTTDYGYQLEMARHITCAAESIVAAEDAPAKIDH

VIRTALREKKPAYLEIACNVAGAPCVRPGGIDALLSPPAPDEASLKAAVD

AALAFIEQRGSVTMLVGSRIRAAGAQAQAVALADALGCAVTTMAAAKSFF

PEDHPGYRGHYWGEVSSPGAQQAVEGADGVICLAPVFNDYATVGWSAWPK

GDNVMLVERHAVTVGGVAYAGIDMRDFLTRLAAHTVRRDATARGGAYVTP

QTPAAAPTAPLNNAEMARQIGALLTPRTTLTAETGDSWFNAVRMKLPHGA

RVELEMQWGHIGWSVPAAFGNALAAPERQHVLMVGDGSFQLTAQEVAQMI

RHDLPVIIFLINNHGYTIEVMIHDGPYNNVKNWDYAGLMEVFNAGEGNGL

GLRARTGGELVAAIEQARANRNGPTLTECTLDRDDCTQELVTWGKRVAAA

NARQPRAG

**> *Gdpdc1.2 (sequence Bertelan et al)***

MTYTVGRYLADRLAQIGLKHHFAVAGDYNLVLLDQLLLNTDMQQIYCSNE

LNCGFSAEGYARANGAAAAIVTFSVGALSAFNALGGAYAENLPVILISGA

PNANDHGTGHILHHTLGTTDYGYQLEMARHITCAAESIVAAEDAPAKIDH

VIRTALREKKPAYLEIACNVAGAPCVRPGGIDALLSPPAPDEASLKAAVD

AALAFIEQRGSVTMLVGSRIRAAGAQAQAVALADALGCAVTTMAAAKSFF

PEDHPGYRGHYWGEVSSPGAQQAVEGADGVICLAPVFNDYATVGWSAWPK

GDNVMLVERHAVTVGGVAYAGIDMRDFLTRLAAHTVRRDATARGGAYVTP

QTPAAAPTAPLNNAEMARQIGALLTPRTTLTAETGDSWFNAVRMKLPHGA

RVELEMQWGHIGWSVPAAFGNALAAPERQHVLMVGDGSFQLTAQEVAQMI

RHDLPVIIFLINNHGYTIEVMIHDGPYNNVKNWDYAGLMEVFNAGEGNGL

GLRARTGGELAAAIEQARANRNGPTLIECTLDRDDCTQELVTWGKRVAAA

NARPPRAG

**> *Gdpdc1.*3 (Sequence Giongo et al)**

MTYTVGRYLADRLAQIGLKHHFAVAGDYNLVLLDQLLLNTDMQQIYCSNE

LNCGFSAEGYARANGAAAAIVTFSVGALSAFNALGGAYAENLPVILISGA

PNANDHGTGHILHHTLGTTDYGYQLEMARHITCAAESIVSAEDAPAKIDH

VIRTALREKKPAYLEIACNVAGAPCVRPGGIDALLSPPAPDEASLKAAVD

AALAFIEQRGSVTMLVGSRIRAAGAQAQAVALADALGCAVTTMAAAKSFF

PEDHPGYRGHYWGEVSSPGAQQAVEGAEGVICLAPVFNDYATVGWSAWPK

GDNVMLVERHAVTVGGVAYAGIDMRDFLTRLAAHTVRRDATARGGAYVTP

QTPAAAPTAPLNNAEMARQIGALLTPRTTLTAETGDSWFNAVRMKLPYGA

RVELEMQWGHIGWSVPAAFGNALAAPERQHVLMVGDGSFQLTAQEVAQMI

RHDLPVIIFLINNHGYTIEVMIHDGPYNNVKNWDYAGLMEVFNAGEGNGL

GLRARTGGELAAAIEQARANRNGPTLIECTLDRDDCTQELVTWGKRVAAA

NARPPRAG

**>*KmPDC5* (NRBC 1777)**

MEYADRYNLEPLIPLAEYLFHRLFQLNCHTVFGVPNYSTAKLYGALAATG

IQWIQTINQLNTSFAADAYGRTIGISCYITSESAELAHINGFFGSYCEYV

PILQLVILEHSHDLERLIGDVSIFHDIVDDPAEIDFGLRTLFWGKRPVYM

GLRSKDISRLVPSIALNQSILNKETPAPFPSSLSLSIARYQQRQQEQSIV

GDIVDQILSKLYSCSTPIIVVDALIDRYNYNDMLQNFLAETGIPFVTTLM

SKAAINESLPNFIGTFLGTLSHPTVREYMNNSDCTLILGCVIDNFKNSYC

RFSYKNKCQIMLWNDRVKIENNLIPDVPIHEILPQLIAKIDASKLSNLYA

VTVPDMVPRVEPKPVTFLRQEYLWFRMSTWLKEGDVIISESGTSAIGLLR

QKFPDNSRLVSQTIWNSSGYSIGACLGVLTAYRDLGKLDKHRIILIVGDG

SLQFTFQELSTILTQGFEPYIYVVNNQGYTVDRTLNKEKTHTNATYFDIQ

QWEILSIPSLFNSRDYFKRKCMTVGELNSLLNDEEFNDPRRLKIVELILP

SMDVPVLLEPHDDSSDDELTPQSKRVRL

**>*ScARO10* (CEN.PK113-7D)**

MAPVTIEKFVNQEERHLVSNRSATIPFGEYIFKRLLSIDTKSVFGVPGDF

NLSLLEYLYSPSVESAGLRWVGTCNELNAAYAADGYSRYSNKIGCLITTY

GVGELSALNGIAGSFAENVKVLHIVGVAKSIDSRSSNFSDRNLHHLVPQL

HDSNFKGPNHKVYHDMVKDRVACSVAYLEDIETACDQVDNVIRDIYKYSK

PGYIFVPADFADMSVTCDNLVNVPRISQQDCIVYPSENQLSDIINKITSW

IYSSKTPAILGDVLTDRYGVSNFLNKLICKTGIWNFSTVMGKSVIDESNP

TYMGQYNGKEGLKQVYEHFELCDLVLHFGVDINEINNGHYTFTYKPNAKI

IQFHPNYIRLVDTRQGNEQMFKGINFAPILKELYKRIDVSKLSLQYDSNV

TQYTNETMRLEDPTNGQSSIITQVHLQKTMPKFLNPGDVVVCETGSFQFS

VRDFAFPSQLKYISQGFFLSIGMALPAALGVGIAMQDHSNAHINGGNVKE

DYKPRLILFEGDGAAQMTIQELSTILKCNIPLEVIIWNNNGYTIERAIMG

PTRSYNDVMSWKWTKLFEAFGDFDGKYTNSTLIQCPSKLALKLEELKNSN

KRSGIELLEVKLGELDFPEQLKCMVEAAALKRNKK

**>*KmARO10* (NRBC 1777)**

MAPVVLDDKSASESPRSDSPVHGLSSVVKDITLGRYVFERLLNCGSKTIF

GVPGDFNLPLLEYLYEEELVQNGLQWVGTCNELNAAYAADGYSRYTSKIG

CVITTFGVGELSALNGISGAFAEDVKVLHIVGVSPTKFRKNDKFRSHNVH

HLVPDLDGDKEPNHEVYFDMIKDRVSCSSAFLHDVESAPEKIDKVIADIY

KYSKPGYIFIPADFADEMVSNKNLVETPVIDLPYVIENTTSKDATKKAGD

KILQWLYESKTPSVFSDALVGRFNLNKDIRELINKVDMWNFTTAMAKSSL

DEHHPKHLGVYKGAETGKEMQSIVEMSDLILHFGPCKNEINFGYYTFRYN

DNARVVELSKDKITFFETKANGSVEVQEANFAAVLKYMNENLDVSKISTA

YPSVSRTREHIEFGEDDEISQQSLKRIVENFYNPGDVLVVETGSFQFNLI

NMKFAPEMKYMTQTFYLSIGMALPAALGVGCGMRDYPRSHIINQSAVPAD

YVPKLILCEGDGAAQMTIQEFASYIRYKIPMNILLFNNNGYTIERAILGP

TRSYNDIAPVKWTALLNAFGDFENKFSETVTVSKNKEIIEVLNEWKKEKV

PSKIKLAEVMLPVMDIPSELDAMLVNGKPK

***>KlARO10 (*CBS 2359)**

MSPVQLDEKSLRTKSPRSDSPVHGQSTVVKNIPFGHYVFERLLNSGAKSI

FGVPGDFNLPLLEYLYDEDLVANGLQWIGTCNELNGAYAADGYSRYTNKL

GCVITTYGVGELSALNGVAGAFAEDVKVLHIIGVSPSRFRENEQFRSHNV

HHLVPAIDSQQPPNHQVYFDMVKDRVCCSSAFLDDINTAADRLDQVIVDI

CKNSKPGYVFIPVDFADKLVSNKNLIQNPIIDMDTVIESYKDENATKIAG

DRLLQWIYESNAPSVFSDLLVDRFNLRKELRQFIETADVWNYNTAMGKST

LDERNPKYLGLYKGAETGKEMQATVDSSDLILHFGPAKNEINCGYYSFHF

NENARIIELSNNIIQFIDIKDKNASTVEEANFVSVLKYVNDNLDKSKLSF

SYPPVPKSNHSCEYDNNDSITQKSLKEIIPNYFNDGDVLIVETGSFQFGV

PNMQFAREMKYITQSFYLSIGMALPAALGVGCGMRDYPRSHIVDQALVPK

DYIPRLILCEGDGAAQMTIQEFASYIRYKIPMEILVWNNNGYTVERAING

PTRSYNDIAPFKWTALLNVFGDFENKFNESVTVEKNDELVQLLNKWKSCK

ERANIKLAEVMLPVMDIPQELNAMLVNGKPN

**>*ScARO10* (S288C)**

MAPVTIEKFVNQEERHLVSNRSATIPFGEYIFKRLLSIDTKSVFGVPGDF

NLSLLEYLYSPSVESAGLRWVGTCNELNAAYAADGYSRYSNKIGCLITTY

GVGELSALNGIAGSFAENVKVLHIVGVAKSIDSRSSNFSDRNLHHLVPQL

HDSNFKGPNHKVYHDMVKDRVACSVAYLEDIETACDQVDNVIRDIYKYSK

PGYIFVPADFADMSVTCDNLVNVPRISQQDCIVYPSENQLSDIINKITSW

IYSSKTPAILGDVLTDRYGVSNFLNKLICKTGIWNFSTVMGKSVIDESNP

TYMGQYNGKEGLKQVYEHFELCDLVLHFGVDINEINNGHYTFTYKPNAKI

IQFHPNYIRLVDTRQGNEQMFKGINFAPILKELYKRIDVSKLSLQYDSNV

TQYTNETMRLEDPTNGQSSIITQVHLQKTMPKFLNPGDVVVCETGSFQFS

VRDFAFPSQLKYISQGFFLSIGMALPAALGVGIAMQDHSNAHINGGNVKE

DYKPRLILFEGDGAAQMTIQELSTILKCNIPLEVIIWNNNGYTIERAIMG

PTRSYNDVMSWKWTKLFEAFGDFDGKYTNSTLIQCPSKLALKLEELKNSN

KRSGIELLEVKLGELDFPEQLKCMVEAAALKRNKK
