# Supplementary material for: Elimination of aromatic fusel alcohols as by-products of Saccharomyces cerevisiae strains engineered for phenylpropanoid production by 2-oxo-acid decarboxylase replacement
Source: Metab Eng Commun. 2021 Sep 7;13:e00183. doi: 10.1016/j.mec.2021.e00183 (PMC8450241; doi:10.1016/j.mec.2021.e00183)

Elimination of aromatic fusel alcohols as by-products of Saccharomyces cerevisiae strains engineered for phenylpropanoid production by 2-oxo-acid decarboxylase replacement

Else-Jasmijn Hassing^1^, Joran Buijs^1^, Nikki Blankerts^1^, Marijke A. Luttik^1^, Erik A.de Hulster^1^, Jack T. Pronk^1^, Jean-Marc Daran^1^*^*^

^1^ Department of Biotechnology, Delft University of Technology, van der Maasweg 9, 2627 HZ Delft, The Netherlands

**Supplementary Figure Legends**

**Figure S1.** **Specific decarboxylase activities for ranging pyruvate concentrations in cell extracts of CENPK711-7C (*pdc1∆ pdc5∆ pdc6∆ aro10∆ thi3∆)* expressing individual 2-oxo acid decarboxylase genes from a multicopy plasmid.** Strains IME419 (*YlPDC1↑*), IME420 (*KmPDC1↑*), IME421 (*ZmPDC1↑*), IME474 (*Gdpdc1.2↑*)*,* IME495 (*Gdpdc1.3↑*), IME615 (*KlPDC1↑*) and IME667 (*ScPDC1↑*) were grown in duplicates at 30 °C on synthetic medium containing 2% (v/v) ethanol as carbon source (SME). Pyruvate concentrations ranged from 0.01 mM to 50 mM. Enzyme activities at each substrate concentration were measured in duplicate. The velocity data were fitted with Michaelis-Menten as well and sigmoidal kinetic model.

**Figure S2 Metabolite profile of the coumaric acid producing reference strain IMX2668 (*Scpdc1Δ, Scpdc5Δ, Scpdc6Δ, Scaro101Δ*) expressing individual 2-OADC genes from a multicopy vector.** IME667 (*ScPDC1↑*), IME658 (*Zmpdc1↑*) ME659 (*KmPDC1↑*), ME660 (*YlPDC1↑*), ME661 (*KlPDC1↑*), IME662 (*Gdpdc1.2↑*) and IME663 (*Gdpdc1.3↑*) were grown at 30 °C in biological triplicates on synthetic medium containing glucose as sole carbon source (SMD). All strains were inoculated at OD_660_ = 0.2 and grown for 72 hours until they had reached stationary phase. Green: Ethanol, white: glucose, blue: pyruvate, orange: glycerol.


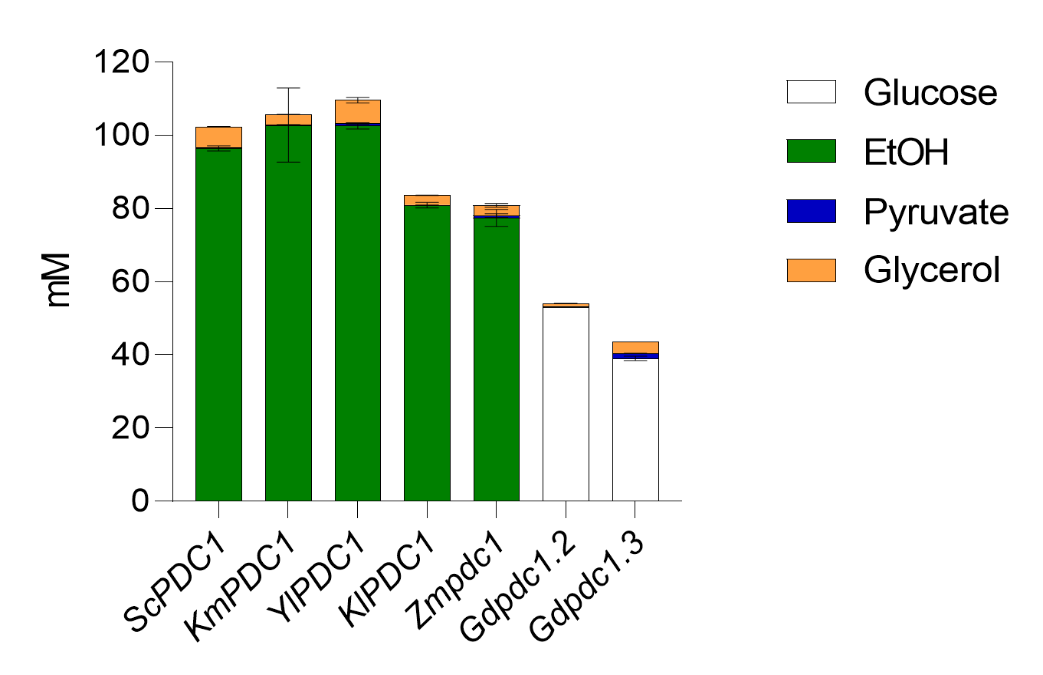

Supplement: Multimedia component 2 [file mmc2.docx]
