# Supplementary material for: Elimination of aromatic fusel alcohols as by-products of Saccharomyces cerevisiae strains engineered for phenylpropanoid production by 2-oxo-acid decarboxylase replacement
Source: Metab Eng Commun. 2021 Sep 7;13:e00183. doi: 10.1016/j.mec.2021.e00183 (PMC8450241; doi:10.1016/j.mec.2021.e00183)
Supplement: Multimedia component 3 [file mmc3.docx]

**Table S.1 All primers used in this study**

| **Plasmid Construction primers** | | |
| --- | --- | --- |
| **Number** | **Sequence** | **Purpose** |
| ASR_A023F | GCATCGTCTCATCGGTCTCAtatgTCTGAAATTACTCTAGGTCGTTA | amplification *KmPDC1* with YTK compatible BsaI and BsmBI restriction flanks -FW |
| ASR_A023Rcorr | ATGCCGTCTCAGGTCTCAGGATCTATTCTTGCTTGGCGTTGAT | amplification *KmPDC1* with YTK compatible BsaI and BsmBI restriction flanks -RV |
| ASR_A024F | GCATCGTCTCATCGGTCTCAtatgGAGTATGCTGATAGGTACAACTT | amplification *KmPDC5* with YTK compatible BsaI and BsmBI restriction flanks -FW |
| ASR_A024MR | TCCGTCTCAacgcTTACCCCAAAACAGCGTT | Removal internal BsaI/BsmBI site *KmPDC5* -RV |
| ASR_A024MF | TTCGTCTCAgcgtCCCGTTTACATGGGTCTT | Removal internal BsaI/BsmBI site *KmPDC5* -FW |
| ASR_A024Rcorr | ATGCCGTCTCAGGTCTCAGGATCTAAAGTCTTACTCTTTTGCTTTGAGG | amplification *KmPDC5* with YTK compatible BsaI and BsmBI restriction flanks -RV |
| ASR_A022F | GCATCGTCTCATCGGTCTCATATGgGCTCCAGTAGTTCTAGACGAT | amplification *KmARO10* with YTK compatible BsaI and BsmBI restriction flanks -FW |
| ASR_A022MR | TTCGTCTCAGTTCttattactaACCatttcatcagcgaaatcagct | Removal internal BsaI/BsmBI site *KmARO10* -RV |
| ASR_A022MF | TTCGTCTCAGAACTTGGTTGAAACACCAGTTATAGACTTGCCTTATGT | Removal internal BsaI/BsmBI site *KmARO10* -FW |
| ASR_A022Rcorr | ATGCCGTCTCAGGTCTCAGGATCTATTTTGGCTTACCATTTACCAACATA | amplification *KmARO10* with YTK compatible BsaI and BsmBI restriction flanks -RV |
| ASR_N009F | GCATCGTCTCATCGGTCTCATATGAAGGCTAAGGACGTT | amplification *CoPlstlA* with YTK compatible BsaI and BsmBI restriction flanks -FW |
| ASR_N009R | ATGCCGTCTCAGGTCTCAGGATTTATTCTTCCAACATAATTTCTGGC | amplification *CoPlstlA* with YTK compatible BsaI and BsmBI restriction flanks -FW |
| 9419 | AAGCATCGTCTCATCGGTCTCAAACGCAATACCAGCCTTCCAACTTC | amplification *FBA1pr* with YTK compatible BsaI and BsmBI restriction flanks -FW |
| 9420 | TTATGCCGTCTCAGGTCTCACATATTTGAATATGTATTACTTGGTTATGG | amplification *FBA1pr* with YTK compatible BsaI and BsmBI restriction flanks -RV |
| 13932 | AAGCATCGTCTCATCGGTCTCATATGGAAACCAAGACCCTGATACAC | amplification *KlPDC5* with YTK compatible BsaI and BsmBI restriction flanks -FW |
| 13933 | CACGTCTCATAGACCAATAGCTGAGGTACCAGATTC | Removal internal BsaI/BsmBI site *KlPDC5* -RV |
| 13934 | TTCGTCTCTTCTACTACAACAAAAATTCCCAGATAA | Removal internal BsaI/BsmBI site *KlPDC5* -FW |
| 13935 | TTATGCCGTCTCAGGTCTCAGGATTCACGTTCTGGGTCTTTTATG | amplification *KlPDC5* with YTK compatible BsaI and BsmBI restriction flanks -RV |
| 13936 | TTATGCCGTCTCAGGTCTCAGGATTTAGTTCTTAGCGTTGGTAG | amplification *KlPDC1* with YTK compatible BsaI and BsmBI restriction flanks -RV |
| 13937 | TTCGTCTCTGAAACCAAGAAGTTGATCGACTTGACTC | Removal internal BsaI/BsmBI site *#2 KlPDC1* -FW |
| 13938 | CACGTCTCATTTCAGCCTTGGCATCGTGTCTGGAAC | Removal internal BsaI/BsmBI site #2 *KlPDC1* -RV |
| 13939 | AAGCATCGTCTCATCGGTCTCATATGTCTGAAATTACATTAGGTCGTTAC | amplification *KlPDC1* with YTK compatible BsaI and BsmBI restriction flanks -FW |
| 13940 | AAGCATCGTCTCATCGGTCTCATATGTCTCCAGTGCAATTAGATGA | amplification *KlARO10* with YTK compatible BsaI and BsmBI restriction flanks -FW |
| 13941 | TTATGCCGTCTCAGGTCTCAGGATTCAATTTGGTTTTCCATTGACTA | amplification *KlARO10* with YTK compatible BsaI and BsmBI restriction flanks -RV |
| 14039 | GCATCGTCTCATCGGTCTCAATCCGGCCCCTTTTCCT | amplification *CYCt* with YTK compatible BsaI and BsmBI restriction flanks -FW |
| 14040 | ATGCCGTCTCAGGTCTCACAGCAAGCTTGTCCCAAAAC | amplification *CYCt* with YTK compatible BsaI and BsmBI restriction flanks -RV |
| 14137 | TTCGTCTCTCGTTTCTTCTCAAGCTAAGCAATTG | Removal internal BsaI/BsmBI site #1 *KlPDC1* -FW |
| 14138 | CACGTCTCAAACGGATGGAACACCGACAAC | Removal internal BsaI/BsmBI site #1 *KlPDC1* -RV |
| 14183 | GCTGAGCATGAGACGGAAATC | amplification pGGKd017 backbone FW |
| 14184 | CGTTCGTCTGCAATTATCGGCCAGTTC | amplification pGGKd017 backbone RV |
| 14185 | CAACGTGGCAATTCGTCGCAATACCGTCTCACTGAACTGGCCGATAATTGCAGACGAACGCGAATATATACTAGCGTTGAATG | *TDH3pr* FW with 60 bp homology to pGGKd017 |
| 14186 | CTTGAATCGGGCAAAGAGATAGTCGCCCAGGTCGACCATTTGGGGTTCGGAGTCGCTCATTTTGTTTGTTTATGTGTGTTTAT | *TDH3pr* RVwith 60 bp homology to *YlPDC1* |
| 14187 | ATGAGCGACTCCGAACCCCAAATG | amplification *YlPDC1* FW |
| 14188 | CTAAACGTTGGTCTTGGCAGAGAG | amplification *YlPDC1* RV |
| 14189 | ATGGACGCGCCTGCCTCGTTGATCAAGCAGGCTGAGCTCTCTGCCAAGACCAACGTTTAGGGCCCCTTTTCCTTTGTCGAT | *CYC1t* FW with 60 bp homology to *YlPDC1* |
| 14190 | CTGTACATGATTCGTCAGTGTGAGCACCACTGACGAGCAGATTTCCGTCTCATGCTCAGCAAGCTTGTCCCAAAACCTTCTC | *CYC1t* RVwith 60 bp homology to pGGKd017 |
| 16851 | AAGCATCGTCTCATCGGTCTCATATGTCTGAAATTACTTTGGGTAAA | amplification *ScPDC1* with YTK compatible BsaI and BsmBI restriction flanks -FW |
| 16852 | TTATGCCGTCTCAGGTCTCAGGATTTATTGCTTAGCGTTGGTAG | amplification *ScPDC1* with YTK compatible BsaI and BsmBI restriction flanks -RV |
| 17630 | AAGCATCGTCTCATCGGTCTCATATGAATTCTAGCTATACACAGAGATATG | amplification *ScTHI3* with YTK compatible BsaI and BsmBI restriction flanks -FW |
| 17631 | TTATGCCGTCTCAGGTCTCAGGATTCAGTATCCAACTTGATTTTTTTTT | amplification *ScTHI3* with YTK compatible BsaI and BsmBI restriction flanks -RV |
| 17825 | GCATCGTCTCATCGGTCTCATATGACCTTACAATCCCAAACTG | amplification *RcTAL1* with YTK compatible BsaI and BsmBI restriction flanks -FW |
| 17826 | ATGCCGTCTCAGGTCTCAGGATTTAGGCTGGAGGGTCTG | amplification *RcTAL1* with YTK compatible BsaI and BsmBI restriction flanks -RV |
| **Cloning primers** | | |
| 4640 | CACCTTTCGAGAGGACGATGCCCGTGTCTAAATGATTCGACCAGCCTAAGAATGTTCAACGATCTACGTATGGTCATTTCTTCTTCAG | amplification *AtC4H and AtCPR1* TU with shrC flank - FW |
| 12040 | GTTGAACATTCTTAGGCTGGTCGAATCATTTAGACACGGGCATCGTCCTCTCGAAAGGTGATTTTTCAAACTGCAAATTCAAG | amplification *CoPlstlA*TU with shrB flank |
| 12044 | ACGTCTCACGGATCGTATATGCCGTAGCGACAATCTAAGAACTATGCGAGGACACGCTAGAACGGCGAATTTTTACTAACC | amplification *CoRcTAL1* TU with shrB flank - RV |
| 18180 | CTAGCGTGTCCTCGCATAGTTCTTAGATTGTCGCTACGGCATATACGATCCGTGAGACGTTCAAGGTGCTCTAATTTTTAAAATTTTTAC | amplification *AtC4H and AtCPR1* TU with shrC flank - R |
| 18181 | AAAAACCAAAACAAAGGCCAAGCACATCGTTTAGAGAAGAAATTTTGGGGGTAATATGTTCAACAGGTGTTGTCCTCTGAG | amplification *CoRcTAL1* TU with 60 bp homology to X3 - RV |
| 18183 | CACAATCCAAGGAAAAATCTGGCCTATATGCAAGGAAGGAGAGATAGTCAAAAGCATTCTGATGAAGTGACGCGCGCCCGGAG | amplification *CoPlstlA*TU with 60 bp homology to X3 - FW |
| **pROS gRNA primers** | | |
| 6178 | GTGCGCATGTTTCGGCGTTCGAAACTTCTCCGCAGTGAAAGATAAATGATCGAAACCGGTACCTCCGCTTTGTTTTAGAGCTAGAAATAGCAAGTTAAAATAAG | *ScPDC1*targetRNA - FW |
| 7246 | TGCGCATGTTTCGGCGTTCGAAACTTCTCCGCAGTGAAAGATAAATGATCATTTACAAGTATTCTAAACCGTTTTAGAGCTAGAAATAGCAAGTTAAAATAAGGCTAGTCCGTTATCAAC | *ScARO10* targetRNA - FW |
| 13614 | TGCGCATGTTTCGGCGTTCGAAACTTCTCCGCAGTGAAAGATAAATGATCATTGTTGTTGCATCATACCTGTTTTAGAGCTAGAAATAGCAAGTTAAAATAAG | *ScPDC5/ScPDC6* targetRNA - FW |
| **Repair Oligo's** | | |
| 7717 | ACTTATTTCACATAATCAATCTCAAAGAGAACAACACAATACAATAACAAGAAGAACAAAGCTAATTAACATAAAACTCATGATTCAACGTTTGTGTATTTTTTTACTTTTGAAGGTTAT | *ScPDC5* knockout repair oligo - FW |
| 7718 | ATAACCTTCAAAAGTAAAAAAATACACAAACGTTGAATCATGAGTTTTATGTTAATTAGCTTTGTTCTTCTTGTTATTGTATTGTGTTGTTCTCTTTGAGATTGATTATGTGAAATAAGT | *ScPDC5* knockout repair oligo - RV |
| 7719 | TCTCAATTATTATCTTCTACTCATAACCTCACGCAAAATAACACAGTCAAATCAATCAAAGCGATTTAATCTCTAATTATTAGTTAAAGTTTTATAAGCATTTTTATGTAACGAAAAATA | *ScPDC1* knockout repair oligo - FW |
| 7720 | TATTTTTCGTTACATAAAAATGCTTATAAAACTTTAACTAATAATTAGAGATTAAATCGCTTTGATTGATTTGACTGTGTTATTTTGCGTGAGGTTATGAGTAGAAGATAATAATTGAGA | *ScPDC1* knockout repair oligo - RV |
| 7247 | ACAAGTTGACGCGACTTCTGTAAAGTTTATTTACAAGATAACAAAGAAACTCCCTTAAGCAAACTTGTGGGCGCAATTATAAAACACTGCTACCAATTGTTCGTTTTCTGTTCATTAACA | *ScARO10* knockout repair oligo - FW |
| 7248 | TGTTAATGAACAGAAAACGAACAATTGGTAGCAGTGTTTTATAATTGCGCCCACAAGTTTGCTTAAGGGAGTTTCTTTGTTATCTTGTAAATAAACTTTACAGAAGTCGCGTCAACTTGT | *ScARO10* knockout repair oligo - RV |
| 7935 | GTGTAGTAGTGATAAACTGGTGCTTCAATTTCTTTTTATGAATTGATCTGTATCTGCACCCATTAGTAGTGTACTCAAAAACGAATTATTGTTGCAAATAAATAAATTTACACAGTTTG | *ScPDC6* knockout repair oligo - FW |
| 7936 | CAAACTGTGTAAATTTATTTATTTGCAACAATAATTCGTTTTTGAGTACACTACTAATGGGTGCAGATACAGATCAATTCATAAAAAGAAATTGAAGCACCAGTTTATCACTACTACAC | *ScPDC6* knockout repair oligo - RV |
| **Diagnostic primers** | | |
| 1504 | GGCGGTAGTGATAACCATTCTC | *ScPDC6* dg primer - RW |
| 1505 | GCCAAAGAGATGAGCCAAAGC | *ScPDC6* dg primer - FW |
| 2012 | GGAAACAGCTATGACCATG | check part plasmids pUD565 - FW |
| 2359 | TGCTTGTACACCTCATGTAG | *ScARO10* dg primer - FW |
| 2360 | GCAGACATTTAGCAGATGTAG | *ScARO10* dg primer - RW |
| 2397 | AGACCGAGATAGGGTTGAGTG | check part plasmids pUD565 - FW |
| 2850 | AGCTGTCCTCGTTGAACATAG | *ScPDC1* dg primer - FW |
| 3065 | ACGTCAAGGCTGAAACTAAG | *ScPDC1* dg - FW |
| 3066 | GATTCAACGGCTTCCTTAAC | *ScPDC1* dg - RV |
| 4240 | AAGCGGACCCAGACTTAAGC | *ScPDC1* dg primer - RW |
| 7722 | CAAAGCGGAGGTACCGGTTTC | *ScPDC1* gRNA dg - RV |
| 7812 | TCATGTAATTAGTTATGTCACGCTTACATTC | *CYC1t* dg primer - FW |
| 8039 | ACTTGAATAATGCAGCGGCG | *PDC5* dg primer - FW |
| 8040 | CACACCACCCTCTTCAATTAGC | *PDC5* dg primer - RW |
| 9901 | ATCTTGTAACCCTTTCCC | *FBA1pr* dg primer - FW |
| 11200 | ATGTTACATGCGTACACGCG | *CYC1t* dg primer - RV |
| 13263 | AGGTATGATGCAACAACAATG | *ScPDC5* and *ScPDC6* gRNA dg - RV |
| 13264 | CGGTTTAGAATACTTGTAAAT | *ScARO10* gRNA dg - RV |
| 13738 | TTAGGGCTTGCGTCAGC | *FBA1pr* dg primer - RV |
| 13944 | TACCATGTCACAGCCCATTG | *KlPDC5* dg primer - FW |
| 13946 | CTTTGGCCAAGCAGACCAAC | *Gdpdc1* dg primer - RV |
| 13947 | TGGCTGCTGCTAAGTCTTTC | *Zmpdc1* dg - FW |
| 13948 | GGATGTCAGTCCAACCAGTA | *Zmpdc1* dg - RV |
| 13951 | AAGATTCGGTGGTGTCTACG | *KlPDC1* dg primer - FW |
| 13952 | GTGGAATTCGACAATGTTCT | *KlPDC1* dg primer - RV |
| 14036 | CTGTGGATAACCGTAGTCGG | check part plasmids pYTK001 - FW |
| 14493 | ACAGAGTTTGAAGCGTATAG | *KmARO10* dg primer - FW |
| 14494 | TTCATTTCTGGAGCGAATTTC | KmARO10 dg primer - RV |
| 14495 | TTGCACATTGTCGGTGTCAG | *KmARO10* dg primer - FW #2 |
| 14496 | TAAACTTCGTGGTTTGGTTC | *KmARO10* dg primer - RV #2 |
| 14497 | TCTGACGCAACAAGCCGATC | *KmPDC5* dg primer - RV |
| 14498 | TCAGACAGGAGTACCTATGG | *KmPDC5* dg primer - FW |
| 14499 | GGTAGGACTATTGGCATAAG | *KmPDC5* dg primer - RV #2 |
| 14500 | AATCGGCACGTACTCGCAAT | *KmPDC5* dg primer - FW #2 |
| 14501 | TGACAGTATCACTCAAAAGA | *KlARO10* dg primer - FW |
| 14502 | GTACACCAAATTGGAAAGAG | *KlARO10* dg primer - RV |
| 14503 | CGGTGTAGCAGGGGCTTTTG | *KlARO10* dg primer - FW #2 |
| 14504 | TGGGAACGGAATTGTTCATT | *KlARO10* dg primer - RV #2 |
| 14506 | TTCTATCGGTGCATGCCTTG | *KlPDC5* dg primer - RV |
| 14507 | TCGCACCGCTAATACCTTTG | *KlPDC5* dg primer - FW #2 |
| 14508 | GGTTACTTGGGGTAAGAGAG | *Gdpdc1* dg primer - FW |
| 14509 | CAACGCTGAAATGGCTAGAC | *Gdpdc1* dg primer - FW #2 |
| 14510 | ATCGCTGAAACTGGTGACTC | *Zmpdc1* dg primer - FW #2 |
| 14511 | TTCTTGCTTGGCGTTGATGG | *KmPDC1* dg primer - FW |
| 14512 | ATGGTTTGGACCTCGACTTG | *KmPDC1* dg primer - RV |
| 14977 | CTCAGTCGAAAGACTGGGCC | check part plasmids pYTK001 - RV |
| 15815 | CAAGATGTGGCGTGTTACGG | check part plasmids pYTK001 - RV |
| 16954 | TTGTCTCCAACCCCAGGTAT | *coPlstA* dg primer - FW |
| 16955 | TGGGGTCAAACCAGCTCTC | *coPlstA* dg primer - RV |
| 17952 | GAATCAGCAAGGCTTAATAC | *ScTHI3* dg primer - FW |
| 17953 | AACCTGTCCACAGAGTAACC | *ScTHI3* dg primer - RV |
| 17954 | TTATGGGTGACGGTGCTTTC | *ScTHI3* dg primer - FW #2 |
| 18090 | TCTAGCTTCGGCCCAAGAC | *coRctal1* dg primer - RV |
