# Supplementary material for: Elimination of aromatic fusel alcohols as by-products of Saccharomyces cerevisiae strains engineered for phenylpropanoid production by 2-oxo-acid decarboxylase replacement
Source: Metab Eng Commun. 2021 Sep 7;13:e00183. doi: 10.1016/j.mec.2021.e00183 (PMC8450241; doi:10.1016/j.mec.2021.e00183)
Supplement: Multimedia component 4 [file mmc4.docx]

**Table S1** **Pyruvate and phenylpyruvate decarboxylase activities in cell extracts of *S. cerevisiae* strain CEN.PK711-7C (*pdc1Δ pdc5Δ pdc6Δ aro10Δ thi3Δ*) expressing individual 2-OADC genes from a multicopy plasmid.** Cell extracts were prepared from late-exponential-phase shake-flask cultures on SMEG. Pyruvate decarboxylase and phenylpyruvate activities were assayed on duplicate cultures.

| Strain | Genotype | Mean V_MAX_  (µmol min^-1^ (mg of protein)^-1^) | |
| --- | --- | --- | --- |
|  |  | Pyruvate | Phenylpyruvate |
| CEN.PK113-7D | wt | 0.17 ± 0.0 | BD |
| IMZ001 | Pdc^-^ | BD | BD |
| IME667 | *ScPDC1* | 8.86 ± 0.5 | 0.098 ± 0.00 |
| IMZ024 | *ScPDC5* | 2.05 ± 0.1 | 0.101 ± 0.01 |
| IMZ031 | *ScPDC6* | 1.31 ± 0.0 | 0.030 ± 0.00 |
| IMZ002 | *ScARO10* | BD | 0.105 ± 0.00 |
| IME420 | *KmPDC1* | 6.20 ± 0.3 | 0.122 ± 0.00 |
| IME423 | *KmARO10* | BD | 0.174 ± 0.00 |
| IME418 | *KlPDC5* | BD | BD |
| IME615 | *KlPDC1* | 5.58 ± 0.3 | 0.103 ± 0.01 |
| IME422 | *KmPDC5* | BD | BD |
| IME424 | *KlARO10* | BD | 0.152 ± 0.01 |
| IME419 | *YlPDC1* | 3.01 ± 0.1 | 0.145 ± 0.00 |
| IME421 | *Zmpdc1* | 8.79 ± 0.5 | BD |
| IME425 | *Gdpdc1.1* | BD | BD |
| IME474 | *Gdpdc1.2* | 0.25 ± 0.0 | BD |
| IME495 | *Gdpdc1.3* | 0.22 ± 0.0 | BD |

BD, below detection limit. For pyruvate decarboxylase activity the detection limit was <0.04 µmol mg of protein^-1^ min^-1^, for phenylpyruvate the limit was at <20 nmol mg of protein^-1^ min^-1^
