# Supplementary figures and images for: Elimination of aromatic fusel alcohols as by-products of Saccharomyces cerevisiae strains engineered for phenylpropanoid production by 2-oxo-acid decarboxylase replacement
Source: Metab Eng Commun. 2021 Sep 7;13:e00183. doi: 10.1016/j.mec.2021.e00183 (PMC8450241; doi:10.1016/j.mec.2021.e00183)

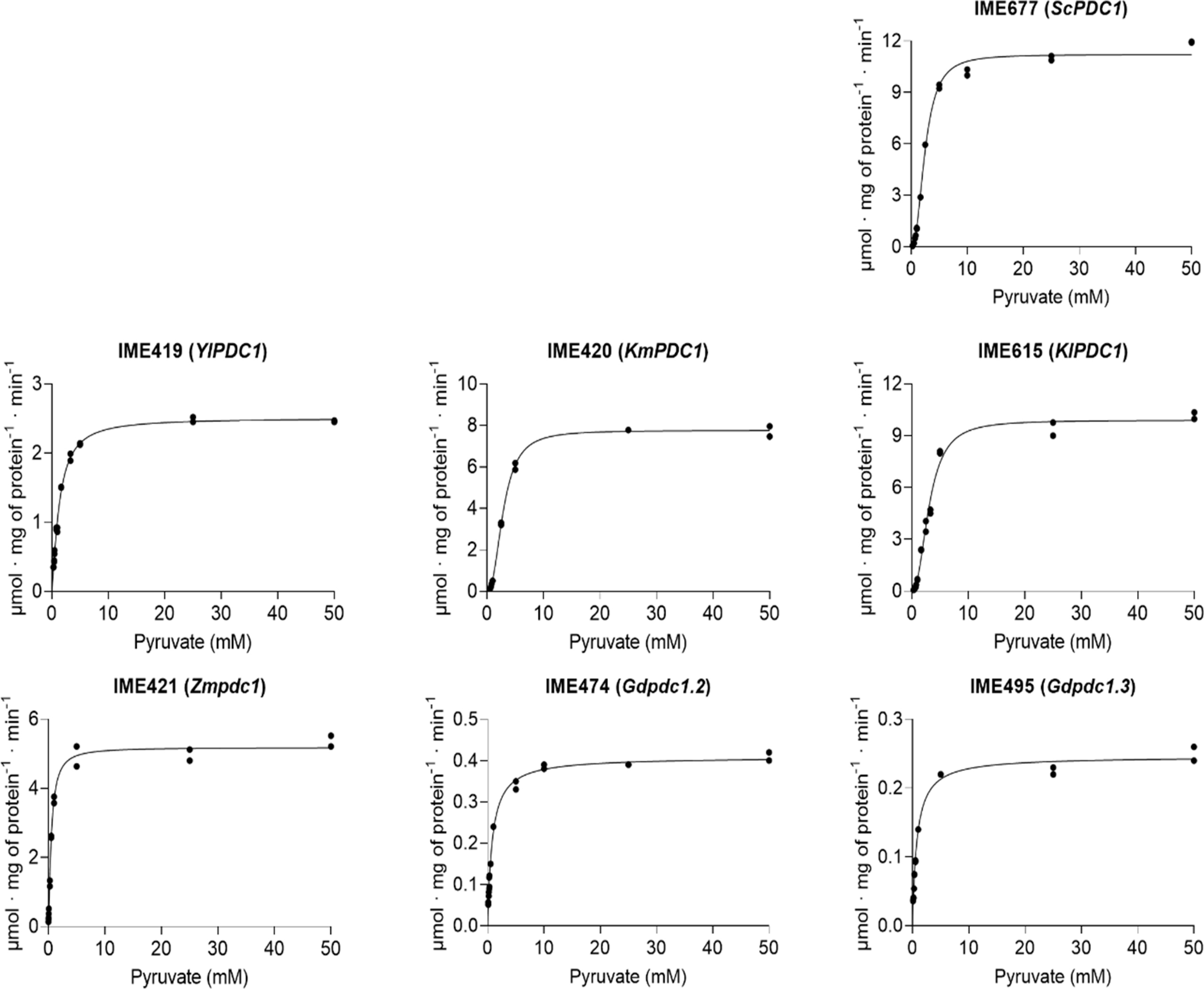

Supplement: figs1 [file mmcfigs1.jpg]

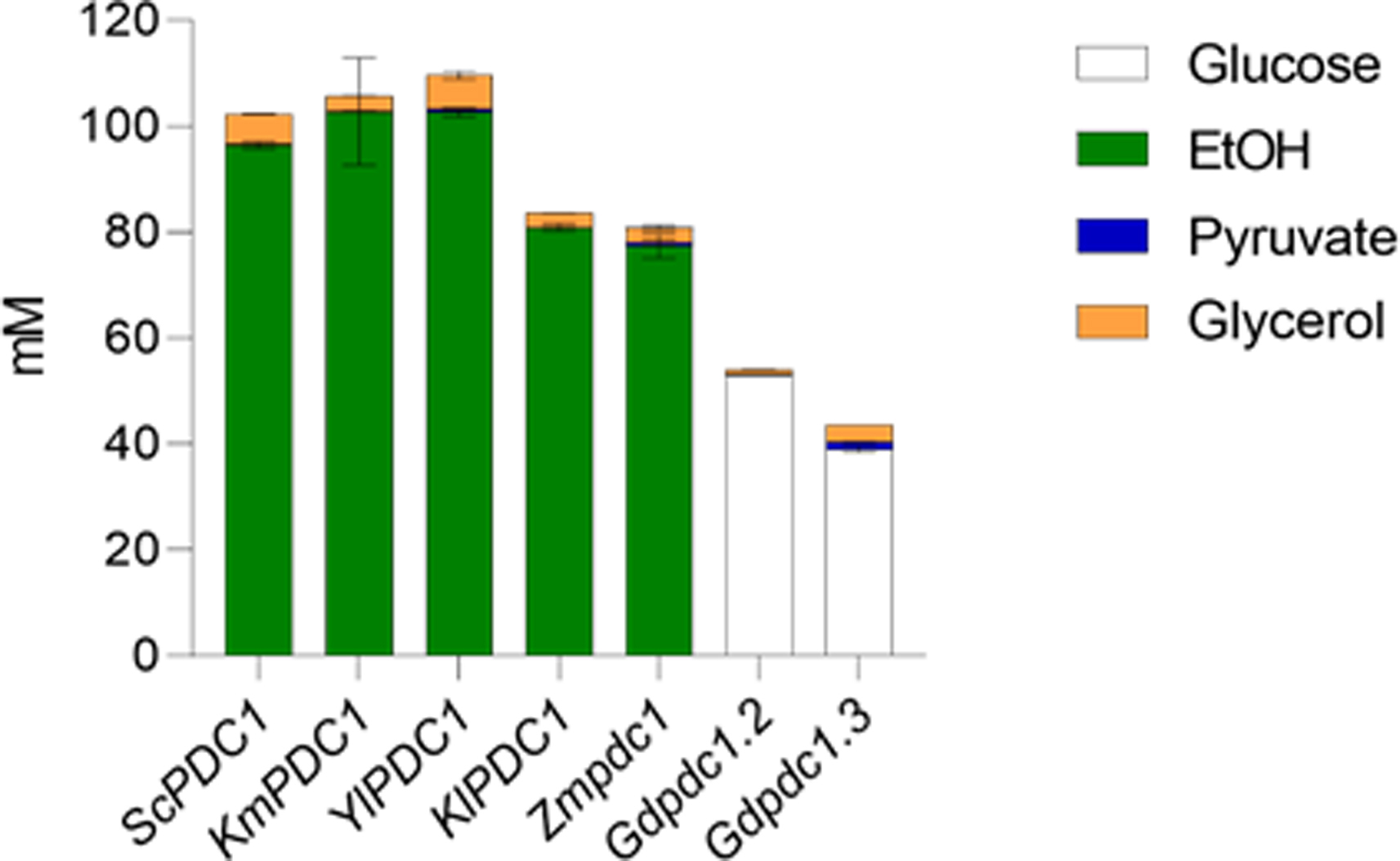

Supplement: figs2 [file mmcfigs2.jpg]
